# Identification of Amino Acid Residues Responsible for C−H Activation in Type‐III Copper Enzymes by Generating Tyrosinase Activity in a Catechol Oxidase

**DOI:** 10.1002/anie.202008859

**Published:** 2020-09-09

**Authors:** Ioannis Kampatsikas, Matthias Pretzler, Annette Rompel

**Affiliations:** ^1^ Universität Wien Fakultät für Chemie Institut für Biophysikalische Chemie Althanstraße 14 1090 Wien Austria

**Keywords:** biotechnology, C−H activation, enzyme engineering, hydroxylase versus oxidase activity, polyphenol oxidases

## Abstract

Tyrosinases (TYRs) catalyze the hydroxylation of phenols and the oxidation of the resulting *o*‐diphenols to *o*‐quinones, while catechol oxidases (COs) exhibit only the latter activity. Aurone synthase (AUS) is not able to react with classical tyrosinase substrates, such as tyramine and l‐tyrosine, while it can hydroxylate its natural substrate isoliquiritigenin. The structural difference of TYRs, COs, and AUS at the heart of their divergent catalytic activities is still a puzzle. Therefore, a library of 39 mutants of AUS from *Coreopsis grandiflora* (*Cg*AUS) was generated and the activity studies showed that the reactivity of the three conserved histidines (HisA_2_, HisB_1_, and HisB_2_) is tuned by their adjacent residues (HisB_1_+1, HisB_2_+1, and waterkeeper residue) either to react as stronger bases or / and to stabilize a position permissive for substrate proton shuffling. This provides the understanding for C−H activation based on the type‐III copper center to be used in future biotechnological processes.

## Graphical abstract

The conserved histidines (HisA_2_, HisB_1_ and HisB_2_), influenced by adjacent residues (HisB_1_+1, HisB_2_+1 and waterkeeper residue), are responsible for the hydroxylase activity. Starting from a catechol oxidase (CO) a tyrosinase (TYR) with monophenolase activity well within the range of naturally occurring plant TYRs has been designed.

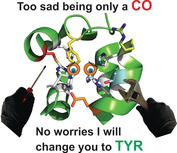

The type‐III copper family includes the enzymes tyrosinase (TYR), catechol oxidase (CO) and aurone synthase (AUS), which are summarized under the umbrella term polyphenol oxidases (PPOs).[^1^, ^2^] They are omnipresent among archaea, bacteria, fungi, animals and plants.[^3^, ^4^, ^5^] TYRs are bi‐functional PPOs catalyzing the *o*‐hydroxylation of monophenols (monophenolase activity, EC 1.14.18.1), which is coupled to the subsequent two‐electron oxidation of the resulting *o*‐diphenols to *o*‐quinones (diphenolase activity, EC 1.10.3.1),^[2]^ whereas COs exhibit only the latter diphenolase reactivity. The enzyme‐in‐between AUS catalyzes *in vivo* the formation of aurones and wild type AUS from *Coreopsis grandiflora* (*Cg*AUS_wt_) exhibits a weak hydroxylase activity towards its natural substrate isoliquiritigenin^[6]^, while it does not react with the classical TYR substrates *l*‐tyrosine and tyramine and was therefore classified as a CO.[^6^, ^7^] PPOs are involved in a wide spectrum of important natural reactions from the browning of fruits^[8]^ over the color of the animals’ skin to being involved in human diseases such as albinism,^[9]^ melanoma^[10]^ and neurodegenerative diseases (Parkinson).^[11]^

The search for the structural difference between TYRs and COs as the basis for their different catalytic activities has been going on for decades. The high similarity of the PPO's active centers from bacteria to animals has been described through 102 PDB entries covering 18 different proteins from 16 organisms (as of July 2020). The discussion about the amino acids decisive for different reactivity is summarized in recent studies.[^12^, ^13^, ^14^, ^15^, ^16^] The initial explanation for the lack of monophenolase activity in COs was the presence of a phenylalanine, the so‐called gatekeeper residue atop of CuA (Figure 1) present in the sweet potato CO (*Ib*CO) structure.[^17^, ^18^] However, the first crystal structure of a plant TYR from *Juglans regia* (*Jr*PPO1) did also show the presence of this bulky phenylalanine, which falsified the idea of the gatekeeper residue as blocking substrate access.^[19]^ A more recent theory claims that the deprotonation of the monophenolic substrate and consequently the tyrosinase activity originates from a highly conserved water molecule (Figure 1) which is stabilized by an asparagine (HisB_1_+1) adjacent to the first CuB coordinating histidine (HisB_1_) and activated by a conserved glutamate, the so‐called waterkeeper residue (Figure 1).^[14]^ However, the asparagine residue at HisB_1_+1 cannot be the solely responsible amino acid, as tyrosinase activity towards the classical substrates has been demonstrated for mushroom *Ab*PPO4,^[20]^ apple (*Md*PPO1 and *Md*PPO3),[^13^, ^21^, ^22^] *Larrea tridentata* PPO^[23]^ and tomato *Sl*PPO1,^[24]^ which all do not contain an asparagine at HisB_1_+1. Recently, two non‐conserved amino acids placed next to the conserved first (HisB_1_+1_;_ Thr253 in *Cg*AUS_wt_) and second histidine (HisB_2_+1_;_ Arg257 in *Cg*AUS_wt_) residues coordinating CuB, have been identified and termed activity controllers, both being located on an α‐helix in front of the PPO's catalytic center (Figure 1).[^12a^, ^13^] Mutagenesis studies on *Jr*PPO1 and *Taraxacum officinale* PPOs (*To*PPO2, a TYR and *To*PPO6, a CO) proved the influence of these amino acid residues on tyrosinase activity.[^15^, ^16^] However, until now no study reported the generation of hydroxylase activity in a CO.

**Figure 1:**
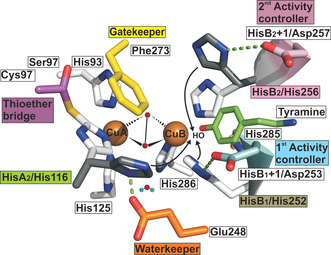
Active center of *Cg*AUS (Cys97Ser/Thr253Asp/Arg257Asp) mutant. The mutated activity controllers Asp253 (cyan, HisB_1_+1), Asp257 (violet, HisB_2_+1), the thioether bridge constituent Cys97 (lilac, Ser97 in the mutant), the gatekeeper residue Phe273 (yellow) and the waterkeeper residue Glu248 (orange) are highlighted. The six conserved histidines His93 (HisA_1_), His116 (HisA_2_) and His125 (HisA_3_) of CuA and His252 (HisB_1_), His256 (HisB_2_), and His286 (HisB_3_) of CuB are depicted (carbon atoms in white and nitrogen atoms in blue). The 7^th^ His285 is depicted (wheat colored) and the water next to Glu248 represents the conserved water modelled into a number of high‐resolution crystal structures.

The numerous published crystal structures of type‐III copper proteins and the considerably smaller number of mutation studies have not yet elucidated the cause for hydroxylase activity in type‐III copper enzymes and thus, a different approach to tackle this puzzling question is necessary. Therefore, we established a library of 39 *Cg*AUS mutants that focus on residues next to the conserved histidines (HisA_2_, HisB_1_ and HisB_2_) which are in close proximity to the substrate, that is, those in front of the active center (Figure 1). Similarly, previous studies converted the activity mode of the investigated enzymes using them as scaffolds (e.g. esterase to aldolase, esterase to epoxide hydrolase and the investigations between phenylalanine and tyrosine ammonia lyases‐mutases).[^25^, ^26^, ^27^] Moreover, mutations targeting the residues gatekeeper (Phe273), waterkeeper (Glu248), the 7^th^ histidine (His285) and two cysteines (Cys31 and Cys32) forming the conserved disulfide bonds (Figures S1 and S2) are summarized in the SI. The *Cg*AUS mutants were heterologously expressed in *E. coli*, purified to homogeneity (Figure S3) and tested towards reactivity with the classical monophenolic substrates tyramine (Figure S4) and l‐tyrosine (Figure S5) and the corresponding diphenols dopamine (Figure S6) and l‐Dopa (Figure S7). For tyramine and dopamine the *K*_m_ and *k*_cat_ values were determined and the mutants’ copper content was measured colorimetrically. The mutants’ ability to form an *oxy*‐adduct has been investigated (Table 1, Table S1). All publicly available crystal structures of type‐III copper enzymes have been taken into consideration to interpret our data.

**Table 1:** Measurements of the copper content (percentage based on 2 copper ions per active center), *k*_cat_ and *K*_m_ values with dopamine and tyramine and the absorption coefficient at *λ*=345 nm after the titration of the mutants with H_2_O_2_. Mutations which failed to create a peak at 345 nm after the incubation with H_2_O_2,_ are presented as “–” and “nd” indicates no detected activity.

Mutants	Copper	Dopamine		Tyramine		H_2_O_2_ϵ_345_
	%	*k*_cat_ [s^−1^]	*K*_m_ [mm]	*k*_cat_ [s^−1^]	*K*_m_ [mm]	[m ^−1^ cm^−1^]
*Cg*AUS_wt_	46.1±1.0	556±27.2	8.63±0.28	nd	nd	5190

Thioether bridge constituent						
Cys97Ala	42.4±1.4	5.84±0.31	1.59±0.09	0.14±0.01	3.03±0.18	–
Cys97Gly	34.1±2.3	43±2.5	7.53±0.45	0.12±0.01	2.96±0.16	–
Cys97Asp	52.7±0.6	1.54±0.07	1.68±0.07	0.07±0.00	1.05±0.02	–
Cys97Asn	45.7±0.6	1.24±0.06	0.80±0.05	0.05±0.00	2.11±0.08	–
Cys97Ser	59.3±0.5	15±1.26	1.26±0.04	0.55±0.04	3.59±0.33	7600

HisB_1_+1 (1^st^ activity controller residue)						
Thr253Asp	45.0±0.9	530±33.7	1.93±0.12	2.14±0.16	30.9±7.27	3800
Thr253Asn	83.6±2.5	850±46.5	4.99±0.38	1.19±0.14	11.5±2.69	19 840
Thr253Glu	46.7±5.2	1394±90.7	3.59±0.22	0.21±0.02	11.5±1.06	7490
Thr253Gly	72.8±0.9	337±24.3	8.74±0.92	0.07±0.00	2.22±0.14	8600
Thr253Ser	52.7±0.0	500±29.0	6.62±0.37	0.01±0.00	1.18±0.09	8570
Thr253Cys	56.9±1.8	312±14.8	11.1±0.33	0.04±0.00	4.32±0.28	14 220
Thr253Ala	56.1±3.2	140±7.2	5.80±0.30	0.05±0.00	1.48±0.04	7850
Thr253Ile	58.2±0.8	27±1.1	13.6±0.72	nd	nd	9980
Thr253Lys	4.8±0.8	20±3.2	38.6±7.88	nd	nd	–

HisB_2_+1 (2^nd^ activity controller residue)						
Arg257Asp	11.1±0.8	1380±95.3	1.26±0.13	8.26±0.48	4.01±0.29	10 470
Arg257Leu	64.5±2.1	2245±125	5.91±0.25	nd	nd	14 890
Arg257Ile	66.3±1.2	1660±93.6	3.57±0.17	nd	nd	11 060
Arg257Gly	79.6±1.8	1264±63.7	3.54±0.26	nd	nd	3490

HisB_1_+1 and HisB_2_+1 (1^st^ and 2^nd^ activity controllers) and thioether bridge constituent						
Thr253Asp Arg257Asp	43.5±1.7	171±10.2	0.24±0.02	9.48±0.55	1.09±0.09	4570
						
Thr253Asp Arg257Gly	32.8±0.4	662±30.0	1.31±0.04	1.91±0.11	4.59±0.25	8610
						
Thr253Gly Arg257Leu	33.8±0.8	535±39.9	4.00±0.46	0.05±0.00	0.75±0.07	7690
						
Thr253Ser Arg257Gly	33.0±0.0	430±26.1	2.37±0.26	0.01±0.00	0.73±0.10	5660
						
Thr253Gly Arg257Val	52.1±0.4	859±52.9	1.29±0.09	0.02±0.00	0.46±0.03	15 990
						
Thr253Gly Arg257Thr	10.6±1.6	191±9.11	2.09±0.09	0.56±0.03	15.5±1.03	2620
						
Cys97Ser Thr253Asp Arg257Asp	40.2±0.6	19±1.22	0.06±0.00	6.52±0.39	0.02±0.00	3700

*Mutations next to the conserved HisB_1_ residue (1* ^*st*^ *activity controller position)*: Nine (Asp, Asn, Glu, Gly, Ser, Cys, Ala, Ile and Lys) mutants have been produced targeting the non‐conserved HisB_1_+1 residue (called 1^st^ activity controller; Thr253 in *Cg*AUS_wt_), and were examined with regard to their hydroxylase activity against tyramine (Table 1 and Figure S4). The tyramine reaction rates of Thr253Asp, Thr253Asn and Thr253Glu featuring either a (deprotonated) carboxylic acid (Asp and Glu) or a carboxamide (Asn) were particularly high (*k*_cat_=2.14, 1.19 and 0.21 s^−1^, respectively) and notably of the same order of magnitude as seen in natural plant TYRs like apple *Md*PPO1‐3 (*k*_cat_=9.5, 0.92 and 1.0 s^−1^, respectively).^[13]^ The mutants Thr253Gly, Thr253Ser, Thr253Cys and Thr253Ala exhibit significantly lower *k*_cat_ values but undoubtedly showed hydroxylase activity, while Thr253Ile and Thr253Lys did not induce monophenolase activity and remained COs (Table 1). The amino acid residues His 252 and its neighbor one 253 are relatively close to each other (2.8 Å in *Cg*AUS_wt_ His252‐Thr253, PDB: 4Z14) due to the turn of the α‐helix (Figure S8). The higher hydroxylase activity in Thr253Asp, Thr253Asn and Thr253Glu is explained by hydrogen bonds which the amino acid residues at position 253 can form with the His252 (HisB_1_+1), and which increase the basicity of the conserved His252. Thus, the incoming monophenolic substrates will be deprotonated by His252 which serves as a base (Figure 2). The faster hydroxylation rate of Thr253Asp over Thr253Glu (2.14 vs. 0.21 s^−1^) indicates the importance of the precise activating amino acid side chain position of the HisB_1_+1 residue. Glutamic acid with its additional CH_2_ group is 4.7 Å away from HisB_1_ showing a weaker hydrogen bond interaction than Asp253 or Asn253 at a distance of 2.6 and 2.8 Å, respectively (Figure S9). Interactions between Asp or Asn with His are similar to catalytic triads as described in the well‐characterized proteases chymotrypsin (PDB: 4H4F) Asp102‐His57‐Ser195 (acid‐base‐nucleophile) and papain (PDB: 1PPN) Asn175‐His159‐Cys25 (stabilizer‐base‐nucleophile, Figure S10).^[28]^ Transient Schiff base formation and glutamate‐supported proton transfer during catalysis have been reported for many reaction mechanisms, for example for aldolases.[^29^, ^30^, ^31^]

**Figure 2:**
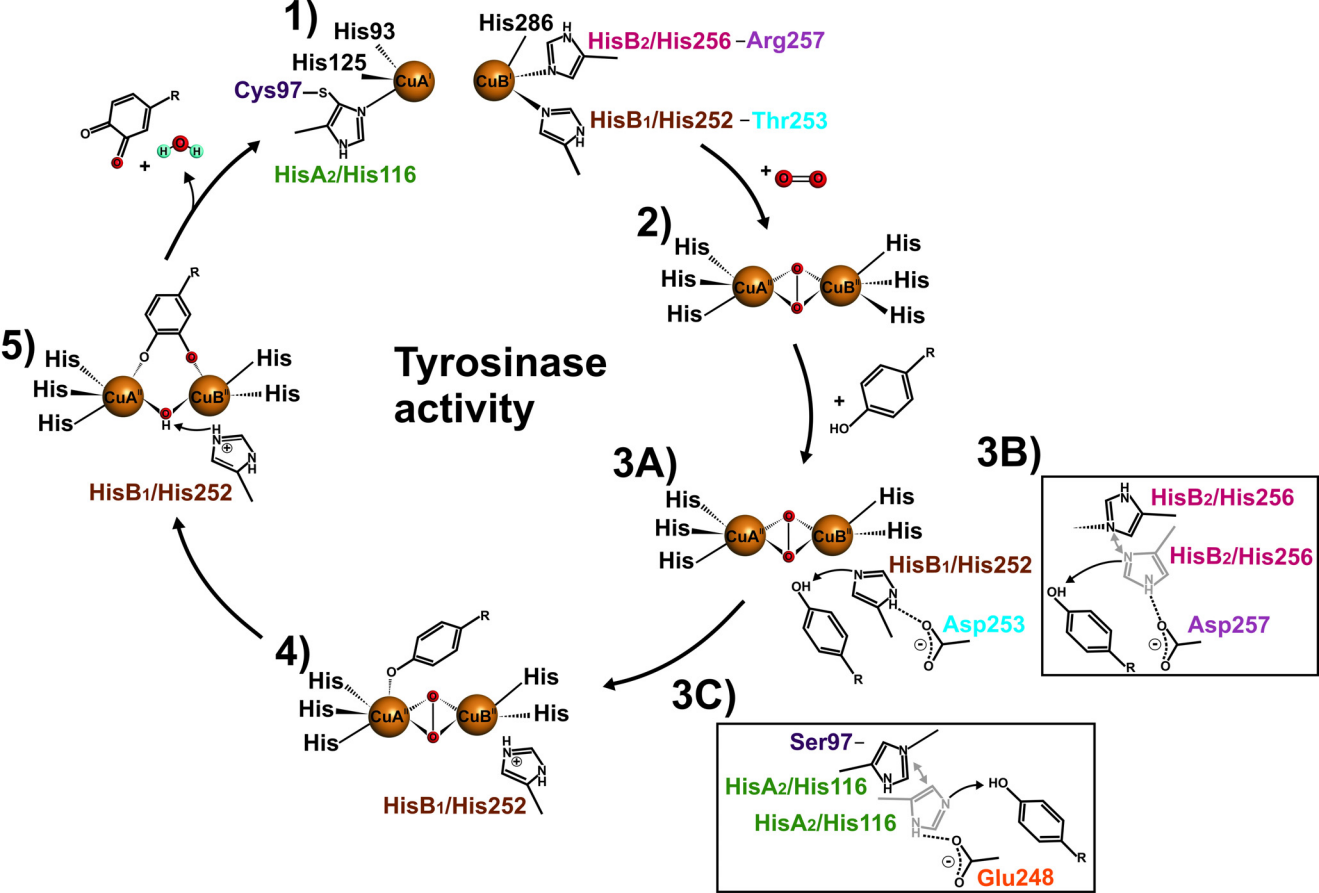
Tyrosinase activity of type‐III copper enzymes. **1)** The deoxy‐form of type‐III copper centers (Cu^I^–Cu^I^) binds molecular oxygen and thereby transitions to the catalytically competent *oxy*‐form (Cu^II^–Cu^II^). **2)** Three of the six conserved histidines (HisA_2_, HisB_1_, and HisB_2_) are responsible for the deprotonation of the incoming monophenolic substrate. **3 A)** In the Thr253Asp mutant HisB_1_′s basicity is increased by the adjacent amino acid residue Asp253 (HisB_1_+1) which can enhance the deprotonation of the incoming substrate. **3 B)** Similarly, in the Arg257Asp mutant HisB_2_ approaches Asp257 (HisB_2_+1) and becomes more basic. **3 C)** In the Cys97Ser mutant HisA_2_ is released by the severing of the thioether bridge and thus is flexible enough to approach the waterkeeper residue Glu248, become more basic and deprotonate the incoming substrate. **4)** The deprotonated monophenol is now susceptible to catalytically productive interaction with the *oxy*‐form of the type‐III copper center. **5)** *Ortho*‐Hydroxylation of the phenolate proceeds via electrophilic aromatic substitution, and subsequent two‐electron oxidation yields an *ortho*‐quinone and water. These two electrons reduce the type‐III copper center to its *deoxy*‐form, thereby closing the catalytic cycle.

*Mutations next to the conserved HisB_2_ residue (2* ^*nd*^ *activity controller position)*: Out of the four mutants (Asp, Leu, Ile and Gly) targeting the HisB_2_+1 (2^nd^ activity controller) residue only Arg257Asp gained tyrosinase activity with an intriguing turnover rate (*k*_cat_=8.26 s^−1^). The generation of monophenolase activity in Arg257Asp is caused by the replacement of the positively charged Arg257 residue in *Cg*AUS_wt_ with the negatively charged Asp257 which enhances the basicity of His256 (HisB_2_) similar to activation of His252 (HisB_1_) by Asp253. However, the side chain of Asp257 is 6.3 Å apart from the conserved His256, and thus, cannot directly influence the basic potential of the conserved His256 from this position. Notwithstanding, the distances mentioned are not to be understood absolutely, as a great deal of flexibility of the two copper ions CuA and CuB, and thus the conserved histidines, has been described several times in PPOs as a prerequisite for functionality.[^32^, ^33^] According to this flexibility of the conserved His, His256 can come closer to Asp257 (2.6 Å) and make a hydrogen bond, forming the charge‐relay network to deprotonate the incoming substrate (Figure S11).

*Double mutants at the HisB_1_+1 and HisB_2_+1 residues (1* ^*st*^ *and 2* ^*nd*^ *activity controllers)*: The Thr253Asp/Arg257Gly, Thr253Gly/Arg257Leu and Thr253Gly/Arg257Thr mutants mimicked the characterized and verified TYRs *Ab*PPO4,^[34]^ *Lt*PPO^[23]^ and *Md*PPO3^[13]^ matching their respective activity controller combinations and all three double mutants are endowed with tyrosinase activity (Table 1). Based on the results obtained for the single mutants at the HisB_1_+1 and HisB_2_+1 residues the double mutant Thr253Asp/Arg257Asp is expected to be the strongest TYR and does indeed show the highest reaction rate on tyramine (*k*_cat_=9.48 s^−1^, Table 1) showing that the negatively charged aspartic acids at the activity controllers’ positions provide so far maximal tyrosinase activity.

*The thioether bridge between the cysteine sulfur and the conserved HisA_2_ residue*: *Cg*AUS_wt_ contains a thioether bridge between the cysteine sulfur (Cys97) and the Cϵ of the conserved HisA_2_ (His116, see Figure 1). This bond fixates the conserved HisA_2_ and is proposed to support electron transfer.[^18^, ^19^] The thioether bridge is a conserved feature in plant and fungal PPOs,[^7^, ^13^, ^33^] whereas it is normally absent in bacteria and mammals (Figure S12).^[14]^ Five mutants (Ala, Gly, Asp, Asn and Ser) that replace Cys97 were produced to prevent thioether bond formation. All five mutants show hydroxylase activity (Table 1), which presumably originates from the release of HisA_2_ from the thioether bridge (Figure 1).^[32]^ Mutants lacking the thioether linkage allow HisA_2_ to approach the entrance of the dicopper center and act as a base and additionally increase its basicity by interacting with the conserved waterkeeper residue Glu248 (Figure 1). The triple mutant Cys97Ser/Thr253Asp/Arg257Asp targeting the three main positions examined in this work (Figure 1), showed a lower activity rate on tyramine (*k*_cat_=6.52 s^−1^) compared to the double mutant Thr253Asp/Arg257Asp (9.48 s^−1^, Table 1) which suggests that HisA_2_ does not add significantly to the deprotonation of the substrate caused by HisB_1_ and HisB_2_. Although not faster, the catalytic efficiency of the mutant Cys97Ser/Thr253Asp/Arg257Asp was ≈326 s^−1^ mm ^−1^ compared to Thr253Asp/Arg257Asp with ≈8.7 s^−1^ mm ^−1^ due to the high specificity of the triple mutant for tyramine (*K*_m_=0.02 mm, Table 1).

*The gatekeeper residue (Phe273), waterkeeper residue (Glu248), 7^th^ His residue (His285), Cys31 and Cys32 residues*: This study also reports on mutants addressing the gatekeeper residue (Phe273), waterkeeper residue (Glu248), 7^th^ His residue (His285), Cys31 and Cys32 residues. Some of them have a minor influence on C−H activation, but exert a significant influence on the total activity of the enzyme, which in most cases is impaired (see SI).

*Flexibility of the conserved histidines facing the substrate as a prerequisite for C−H activation in type‐III copper centers*: In PPOs, the flexibility of the two copper ions is high as supported by crystallographic data obtained from different enzymatic stages (*oxy*, *met* and *deoxy*). The structures of *oxy* (PDB: 4Z13) and *deoxy* (PDB: 4Z14) *Cg*AUS exhibit a difference of ≈1 Å in the copper‐copper distance. Upon formation of the *oxy*‐form both copper atoms move from the initial *deoxy* copper position, CuA by 0.5 Å and CuB by 0.7 Å.^[7]^ Recently, Matoba *et* *al*. investigated the bacterial TYR from *Streptomyces castaneoglobisporus* and showed that the flexibility of the two Cu ions has an impact on the position of the conserved histidines.^[32]^ Moreover, Fujieda *et* *al*. show a similar influence of copper ion flexibility on conserved His of *Aspergillus oryzae* TYR and based on crystallographic studies proposed that the deprotonation of the substrate probably happens by HisA_3_ (His103) because of its lost interactions with CuA during substrate approach.^[35]^ Furthermore, the crystal structure of active mushroom TYR *Ab*PPO4^[33]^ revealed two CuB conformations with 2.3 Å distance between them. The second CuB position shows interaction with a fourth imidazole group (three with the conserved histidines and one additional with the 7^th^ histidine). Therefore, in *Ab*PPO4, the 7^th^ histidine can react as a backup residue and supports the flexibility of the copper ion and histidines.^[33]^ All of this reveals that PPOs contain a flexible dicopper center where the two copper ions can occupy different positions and consequently the conserved histidines are available for the additional task of substrate deprotonation besides copper coordination.

*Formation of the oxy‐complex as the primary step in the monophenolase cycle*: All *Cg*AUS mutants were titrated with H_2_O_2_ and their conversion to the *oxy*‐form was investigated by determining the ϵ_345_ absorption coefficient (Table 1 and S1) and the number of equivalents needed for saturation (Table S1). All mutants targeting the HisB_1_+1 and HisB_2_+1 (Thr253 and Arg257) residues which exhibited monophenolase activity did also form an *oxy*‐adduct. Mutants Thr253Ile, Arg257Leu and Arg257Ile were unable to hydroxylate monophenolic substrates although showing a strong *oxy*‐adduct induced by H_2_O_2_ (Table S1). Hence, the ability of PPOs to form a stable *oxy*‐adduct represents merely the first step in the monophenolase cycle (Figure 2).

*Monophenolase cycle for type‐III copper enzymes (Figure* 2 *)*: *Deoxy‐Cg*AUS (Cu^I^) binds molecular oxygen and forms the *oxy*‐form (Cu^II^) **(1→2)**. The incoming monophenolic substrate needs to be deprotonated, which is supported by one of the three conserved histidines HisA_2_, HisB_1_ and HisB_2_ *en route* to the active *oxy*‐site **(2→3 A**, **3 B** and **3 C)**. Due to the flexibility of the two copper ions the detached histidines serve as bases **(3)**. The reactivity of the three histidines (HisA_2_, HisB_1_ and HisB_2_) is tuned by their adjacent residues (HisB_1_+1, HisB_2_+1 and waterkeeper residue) either to react as stronger bases or / and to stabilize a position suitable for substrate proton shuffling **(3)**. The deprotonated substrate binds to CuA^[32]^ or in the middle of the two copper ions^[7]^ resulting in hydroxylation **(4)**. The dicopper center converts to the *met*‐form after transferring one oxygen atom to the substrate **(5)** and the oxidation of the diphenolic substrate leads to the final quinone product while the enzyme returns to the *deoxy*‐form for a new catalytic cycle **(5→1)**.

*Implications*: Firstly, the residues that control the substrate specificity in type‐III copper proteins have been identified. The type of amino acids present at these positions causes the disparity of the structurally similar TYRs and COs. Secondly, we demonstrated how a CO type enzyme can be converted to a TYR by mutating the residues HisB_1_+1, HisB_2_+1 or the Cys of the thioether bridge. Finally, our findings contribute to the basic understanding of the monophenolase reaction cycle (Figure 2). The present study explains for the first time that hydroxylase activity in type‐III copper enzymes is a result of the flexibility of the three conserved HisA_2_, HisB_1_ and HisB_2_, allowing them to act as bases and deprotonate monophenolic substrates initiating the C−H activation in TYRs, in contrast to COs. The control of the C−H activation reaction will fundamentally impact several important applications in the fields of medicine (melanoma), biotechnology, bioremediation, post‐harvest technology, textile technology, wine production and others which are of utmost importance.

## Conflict of interest

The authors declare no conflict of interest.

## Supporting information

As a service to our authors and readers, this journal provides supporting information supplied by the authors. Such materials are peer reviewed and may be re‐organized for online delivery, but are not copy‐edited or typeset. Technical support issues arising from supporting information (other than missing files) should be addressed to the authors.

Supplementary

## References

[1] E. I. Solomon , D. E. Heppner , E. M. Johnston , J. W. Ginsbach , J. Cirera , M. Qayyum , M. T. Kieber-Emmons , C. H. Kjaergaard , R. G. Hadt , L. Tian , Chem. Rev. 2014, 114, 3659–3853. 24588098 10.1021/cr400327t PMC4040215

[2] C. Kaintz , S. G. Mauracher , A. Rompel , in Adv. Protein Chem. Struct. Biol. (Ed.: Christov C. Z.), Academic Press, San Diego, 2014, pp. 1–35. 10.1016/bs.apcsb.2014.07.001 25458353

[3] A. M. Mayer , Phytochemistry 2006, 67, 2318–2331. 16973188 10.1016/j.phytochem.2006.08.006

[4] C. Kaintz , C. Molitor , J. Thill , I. Kampatsikas , C. Michael , H. Halbwirth , A. Rompel , FEBS Lett. 2014, 588, 3417–3426. 25109778 10.1016/j.febslet.2014.07.034 PMC4158910

[5] M. Pretzler , A. Bijelic , A. Rompel , in Ref. Module Chem. Mol. Sci. Chem. Eng., Elsevier, Amsterdam, 2015, 10.1016/B978-0-12-409547-2.11521-5.

[6] C. Molitor , S. G. Mauracher , S. Pargan , R. L. Mayer , H. Halbwirth , A. Rompel , Planta 2015, 242, 519–537. 25697287 10.1007/s00425-015-2261-0 PMC4540782

[7] C. Molitor , S. G. Mauracher , A. Rompel , Proc. Natl. Acad. Sci. USA 2016, 113, E1806–E1815. 26976571 10.1073/pnas.1523575113 PMC4822611

[8] A. Derardja , M. Pretzler , I. Kampatsikas , M. Barkat , A. Rompel , Food Chem. X 2019, 4, 100053. 31650127 10.1016/j.fochx.2019.100053 PMC6804514

[9] M. B. Dolinska , N. Kus , K. Farney , P. T. Wingfield , B. P. Brooks , Y. V. Sergeev , Pigment Cell Melanoma Res. 2017, 30, 41–52. 27775880 10.1111/pcmr.12546 PMC5568694

[10] J. L. Boyle , H. M. Haupt , J. B. Stern , H. A. B. Multhaupt , Arch. Pathol. Lab. Med. 2002, 126, 816–822. 12088451 10.5858/2002-126-0816-TEIMMD

[11] A. Bose , G. A. Petsko , D. Eliezer , J. Parkinson′s Dis. 2018, 8, 385–398. 29991141 10.3233/JPD-171263 PMC6130416

[12a] M. Pretzler , A. Rompel , Inorg. Chim. Acta 2018, 481, 25–31;

[12b] H. Decker , E. Solem , F. Tuczek , Inorg. Chim. Acta 2018, 481, 32–37.

[13] I. Kampatsikas , A. Bijelic , M. Pretzler , A. Rompel , Sci. Rep. 2017, 7, 8860. 28821733 10.1038/s41598-017-08097-5 PMC5562730

[14] M. Goldfeder , M. Kanteev , S. Isaschar-Ovdat , N. Adir , A. Fishman , Nat. Commun. 2014, 5, 4505. 25074014 10.1038/ncomms5505

[15] S. M. Prexler , M. Frassek , B. Moerschbacher , M. E. Dirks-Hofmeister , Angew. Chem. Int. Ed. 2019, 58, 8757–8761; 10.1002/anie.201902846 31037807

[16] F. Panis , I. Kampatsikas , A. Bijelic , A. Rompel , Sci. Rep. 2020, 10, 1659. 32015350 10.1038/s41598-020-57671-x PMC6997208

[17] K. A. Magnus , B. Hazes , H. Ton-That , C. Bonaventura , J. Bonaventura , W. G. J. Hol , Proteins Struct. Funct. Bioinf. 1994, 19, 302–309. 10.1002/prot.340190405 7984626

[18] T. Klabunde , C. Eicken , J. C. Sacchettini , B. Krebs , Nat. Struct. Mol. Biol. 1998, 5, 1084–1090. 10.1038/4193 9846879

[19] A. Bijelic , M. Pretzler , C. Molitor , F. Zekiri , A. Rompel , Angew. Chem. Int. Ed. 2015, 54, 14677–14680; 10.1002/anie.201506994 PMC4678486 26473311

[20] S. G. Mauracher , C. Molitor , C. Michael , M. Kragl , A. Rizzi , A. Rompel , Phytochemistry 2014, 99, 14–25. 24461779 10.1016/j.phytochem.2013.12.016 PMC3969299

[21] I. Kampatsikas , A. Bijelic , M. Pretzler , A. Rompel , Acta Crystallogr. Sect. F 2017, 73, 491–499. 10.1107/S2053230X17010822 PMC5544008 28777094

[22] I. Kampatsikas , A. Bijelic , M. Pretzler , A. Rompel , Angew. Chem. Int. Ed. 2019, 58, 7475–7479; 10.1002/anie.201901332 PMC6563526 30825403

[23] H. J. Martin , I. Kampatsikas , R. Oost , M. Pretzler , E. Al-Sayed , A. Roller , G. Giester , A. Rompel , N. Maulide , Chem. Eur. J. 2018, 24, 15756–15760. 30113748 10.1002/chem.201803785 PMC6220842

[24] I. Kampatsikas , A. Bijelic , A. Rompel , Sci. Rep. 2019, 9, 4022. 30858490 10.1038/s41598-019-39687-0 PMC6411738

[25] M. Svedendahl , K. Hult , P. Berglund , J. Am. Chem. Soc. 2005, 127, 17988–17989. 16366534 10.1021/ja056660r

[26] H. Jochens , K. Stiba , C. Savile , R. Fujii , J.-G. Yu , T. Gerassenkov , R. J. Kazlauskas , U. T. Bornscheuer , Angew. Chem. Int. Ed. 2009, 48, 3532–3535; 10.1002/anie.200806276 19350592

[27] S. Bartsch , U. T. Bornscheuer , Angew. Chem. Int. Ed. 2009, 48, 3362–3365; 10.1002/anie.200900337 19343746

[28] T. Klein , U. Eckhard , A. Dufour , N. Solis , C. M. Overall , Chem. Rev. 2018, 118, 1137–1168. 29265812 10.1021/acs.chemrev.7b00120

[29] C. Zeymer , R. Zschoche , D. Hilvert , J. Am. Chem. Soc. 2017, 139, 12541–12549. 28783336 10.1021/jacs.7b05796

[30] V. Sautner , M. M. Friedrich , A. Lehwess-Litzmann , K. Tittmann , Biochemistry 2015, 54, 4475–4486. 26131847 10.1021/acs.biochem.5b00283

[31] L. Giger , S. Caner , R. Obexer , P. Kast , D. Baker , N. Ban , D. Hilvert , Nat. Chem. Biol. 2013, 9, 494–498. 23748672 10.1038/nchembio.1276 PMC3720730

[32] Y. Matoba , S. Kihara , N. Bando , H. Yoshitsu , M. Sakaguchi , K. Kayama , S. Yanagisawa , T. Ogura , M. Sugiyama , PLOS Biol. 2018, 16, e3000077. 30596633 10.1371/journal.pbio.3000077 PMC6312201

[33] S. G. Mauracher , C. Molitor , R. Al-Oweini , U. Kortz , A. Rompel , Acta Crystallogr. Sect. D 2014, 70, 2301–2315. 25195745 10.1107/S1399004714013777 PMC4157443

[34] M. Pretzler , A. Bijelic , A. Rompel , Sci. Rep. 2017, 7, 1810. 28500345 10.1038/s41598-017-01813-1 PMC5431950

[35] N. Fujieda , K. Umakoshi , Y. Ochi , Y. Nishikawa , S. Yanagisawa , M. Kubo , G. Kurisu , S. Itoh , Angew. Chem. Int. Ed. 2020, 59, 13385–13390; 10.1002/anie.202004733 32356371

