# Supplementary material for: Identification of Amino Acid Residues Responsible for C−H Activation in Type‐III Copper Enzymes by Generating Tyrosinase Activity in a Catechol Oxidase
Source: Angew Chem Int Ed Engl. 2020 Sep 9;59(47):20940–5. doi: 10.1002/anie.202008859 (PMC7693034; doi:10.1002/anie.202008859)
Supplement: Supplementary file 1 — Supplementary [file ANIE-59-20940-s001.pdf]

Angewandte Chemie
Eine Zeitschrift der Gesellschaft Deutscher Chemiker

Supporting Information

Identification of Amino Acid Residues Responsible for C–H Activation in Type-III Copper Enzymes by Generating Tyrosinase Activity in a Catechol Oxidase

*Ioannis Kampatsikas, Matthias Pretzler, and Annette Rompel**

anie_202008859_sm_miscellaneous_information.pdf

Author Contributions

I.K. Conceptualization: Lead; Data curation: Lead; Writing - Review & Editing: Lead
M.P. Conceptualization: Supporting; Data curation: Supporting; Writing - Review & Editing: Supporting
A.R. Funding acquisition: Lead; Project administration: Lead; Supervision: Lead; Writing - Original Draft: Supporting; Writing - Review & Editing: Supporting.

Supporting Information

Table of Contents

1. Supplementary Material and Methods 2

2. Supplementary Notes 7

2. Supplementary Figures 9

3. Supplementary Tables 29

4. References 33

1. Supplementary Material and Methods

Mutations around the catalytic dicopper center of *Cg*AUS. The gene of the pro-*Cg*AUS$_{wt}$ cloned in the expression vector pGEX-6P-1[1] was used as template for the construction of the *Cg*AUS-mutants (Table S1) and the heterologous expression in *Escherichia coli*. Primers have been designed (Table S2) and a site directed mutagenesis method (Q5® Site-Directed Mutagenesis Kit NEB) was used. The majority of the mutants were targeting the five residues Thr253 (HisB$_1$+1 or 1st activity controller residue), Arg257 (HisB$_2$+1 or 2nd activity controller residue), Cys97 (thioether bridge constituent, linked with His116), Phe273 (gatekeeper residue) and Glu248 (waterkeeper residue, Table S1). All these mutants (35 targeting these 5 residues), as well as the mutations targeting the 7thHis (His285Ala) and the one deleting the loop (Asn98-Ile115) next to the thioether bridge were heterologously expressed as soluble proteins and purified to homogeneity (yield of ~ 10-30 mg per liter of culture, Figure S3).

Cloning, heterologous expression in *E. coli* and purification of the *Cg*AUS mutants. Cloning of *Cg*AUS in the pGEX-6P-1 vector has already been described elsewhere.[1] The *Cg*AUS mutant genes were N-terminally fused with the GST-tag of the pGEX-6P-1 vector. The human rhinovirus 3C protease (HRV3C) recognition sequence (LEVLFQ|GP) was located between the two fusion partners enabling the controlled proteolytic dissociation of the two proteins. The fusion genes (GST-*Cg*AUS) were efficiently overexpressed using the synthetic *tac* promoter of the pGEX-6P-1 vector. *E. coli* was grown in a modified 2xYT medium (1.6 % tryptone-peptone, 1 % yeast extract, 1 % NaCl, 0.5 % NH_4Cl, 0.5 % glycerol, 2 mM $MgCl_2$, 1 mM $CaCl_2$ at pH 7.5) supplemented with ampicillin (100 µg/ml). The expression batches were inoculated with saturated overnight cultures and grown at 37 °C under shaking for 4 hours until the OD_{600} reached a value between 0.6 and 0.8. Afterwards, the temperature of the *Cg*AUS containing batches was reduced to 25 °C. The expression was induced with 0.5 mM isopropyl β-D-1-thiogalactopyranoside (IPTG) and 0.5 mM $CuSO_4$ was also added. The expression cultures remained under shaking at 25 °C for 24 hours. The cultures were then collected by centrifugation at 6000 x g for 25 minutes at 4 °C. Lysis of the cells was effectuated by the freeze-thaw technique using liquid nitrogen. The pellets were re-suspended in lysis buffer (50 mM Tris-HCl pH 7.5, 200 mM NaCl, 1 mM EDTA and 50 mM sucrose). Lysozyme (0.5 g/l) and protease inhibitors (1 mM phenylmethylsulfonyl fluoride and 1 mM benzamidine) were added and the resulting suspensions were incubated for 45 minutes under shaking on ice. Subsequently, the solutions underwent five cycles of freezing in liquid nitrogen and thawing in a water bath at 25 °C. Eventually, 2 mM $MgCl_2$ and 0.02 g/l DNase I were added to the lysates, which were then incubated at 25 °C and 100 rpm for 15 minutes. The lysates were centrifuged at 7000 x g for 1 hour at 4 °C. The chromatographic

purifications were carried out using an Äkta Purifier (GE Healthcare) placed in a refrigerator at 4 °C. The filtrated lysates were placed in a 50 ml injection loop and were applied to a pre-packed 5 ml GSTrap FF column using 50 mM Tris-HCl pH 7.5 and 200 mM NaCl as the binding buffer. Following the trapping and flushing out of unbound proteins, the target proteins were eluted with 50 mM Tris-HCl pH 7.5, 200 mM NaCl and 15 mM reduced glutathione. Fractions containing the GST-fusion protein were pooled and concentrated using a Vivaspin ultrafiltration device with a 30 kDa molecular weight cut-off (VWR). The buffer was then exchanged to 50 mM Tris-HCl pH 7.0, 200 mM NaCl, 1 mM EDTA and the samples were mixed with GST-HRV3C, which was produced in-house[2] at a mass ratio of 1:75 (protease : fusion protein). The proteolysis was carried out over 48 hours at 4 °C. The cleaved protein was then again applied to the 5 ml GSTrap FF column, whereby the GST protein and the GST-tagged protease were still trapped by the column while the latent *Cg*AUS mutants passed through the column and were immediately eluted in the flow-through. Subsequently, the enzymes were concentrated and stored in 50 mM Tris-HCl pH 7.0. The protein concentrations were determined according to the Lambert-Beer law and their absorption at 280 nm using the extinction coefficient provided by ExPASy ProtParam.[3,4]

SDS-PAGE analysis and determination of protein concentration. The quality of heterologous expression and the purity of the recombinant proteins were analyzed *via* SDS-PAGE using 13 % acrylamide gels.[5] The samples were mixed with half a volume of loading buffer (250 mM Tris-HCl pH 6.8, 3 % SDS, 20 % glycerol, 0.02 % bromophenol blue and 7.5 % (v/v) β-mercaptoethanol) and were denatured at high temperature (99 °C) for 5 minutes. Afterwards, the samples were loaded on the SDS-PAGE gel and run in a Mini-PROTEAN Tetra Cell System (Biorad, Vienna, Austria). Precision Plus Protein Dual Color Standard (Biorad) was used as the molecular weight marker. The separated proteins were visualized by Coomassie staining (0.02 % Coomassie brilliant blue G-250, 5 % aluminium sulfate, 10 % ethanol and 2 g/L *ortho*-phosphoric acid) and the gels were destained in 10 % ethanol and 20 g/L *ortho*-phosphoric acid.

Proteolytic activation of pro-*Cg*AUS. The activation of pro-PPO to the active enzyme has been described before for apple PPO (*Md*PPO2)[6] and *Ab*PPO4 from *Agaricus bisporus*.[2] Conversion of pro-*Cg*AUS into the active enzyme has been tested by treatment with three different serine proteases (Trypsin, Proteinase K and Nagarse). The proteolytic digestion of pro-*Cg*AUS was checked applying different ratios of protease and *Cg*AUS (µg protease : µg *Cg*AUS): 1:240 for 1 min, 1:240 for 2 min, 1:240 for 4 min, 1:240 for 10 min, 1:120 for 1 min, 1:120 for 2 min, 1:120 for 3 min and 1:120 for 4 min. However, homogeneity of the active enzyme was not attained by treating pro-*Cg*AUS with any of the investigated proteases (Figure S13) and therefore, the pro-enzymes of the investigated mutants were used in the

kinetic measurements with the use of 1.5 mM sodium dodecyl sulfate (SDS) as activator. This is a classical method to activate pro-PPOs and it has been proven that the generated activity is similar to the activity of the active enzyme.[6]

Copper content determination: The copper content was measured for *Cg*AUS$_{wt}$ and the investigated *Cg*AUS-mutants. The copper ions of 600 µg purified enzyme were reduced to Cu^{I} with 50 mM sodium ascorbate and were then diluted to a total volume of 400 µl with 100 mM sodium phosphate buffer at pH 6.0. Subsequently 600 µl of a 0.5 g/l 2,2'-biquinoline solution in glacial acetic acid were added to a final volume of 1 ml. The solution was incubated for 10 minutes and the formation of the copper-2,2'-biquinoline complex was measured at 546 nm (ε = 6300 M^{-1} cm^{-1}),[7] all measurements were applied in triplicates. The copper content varies notably in different mutants (Table S1). Thr253Asn, Arg257Asp and Phe273Leu exhibit copper content of 83.5 %, 79.6 % and 89.5 % respectively, whereas the copper content was zero in Glu248Lys and the loop deletion (Asn98-Ile115) mutant. It is important to note that a high copper content does not necessarily equal monophenolase activity as exemplified by Thr253Ile, Arg257Leu, Arg257Ile which all show more than 50 % of copper but did not exhibit monophenolase activity while Thr253Asp/Arg257Asp shows the highest monophenolase activity with 43.5 % copper (Table S1).

Enzyme kinetics and substrate activity assays. The activity was determined spectrophotometrically by detecting the appearance of the chromophoric quinones at 480 nm, which are produced by the reaction of the substrates (monophenols: tyramine, *L*-tyrosine and diphenols: dopamine and *L*-3,4-dihydroxyphenylalanine (*L*-DOPA)) with *Cg*AUS$_{wt}$ and the investigated mutants in the presence of molecular oxygen. Absorption curves and spectra were recorded at 25 °C in a 96 well microplate applying a TECAN infinite M200 (Tecan). Kinetic measurements were performed in a total volume of 200 µl, containing 50 mM Tris-HCl buffer (pH 7.0), different molarities of one of two substrates (tyramine and dopamine) and different molarities of the investigated enzymes in the presence of 1.5 mM SDS for activation of the inactive pro-enzymes. Moreover, the acceptance of the monophenols tyramine and *L*-tyrosine and the diphenols dopamine and *L*-DOPA were investigated for all the mutants by substrate acceptance assays (Figures S4, S5, S6 and S7). For tyramine and dopamine the molar absorption coefficients ($\varepsilon_{\lambda max}$) of the formed chromophores (3300 M^{-1} cm^{-1}) at 480 nm have already been reported before.[8]

Formation of the *oxy*-adduct. *Cg*AUS$_{wt}$ and *Cg*AUS-mutants were spectrophotometrically examined using H_2O_2 (Figures S14, S15, S16, S17, S18 and S19). Addition of H_2O_2 to type-III copper enzymes like *Cg*AUS leads to the formation of a new absorption band around ~ 345 nm which is characteristic for the oxygen induced *oxy*-form of the dicopper center. In

these experiments 1 mg of the investigated enzymes (*Cg*AUS_{wt} and *Cg*AUS-mutants) were mixed into a 600 µl solution of the storage buffer (50 mM Tris-HCl and 200 mM NaCl at pH 7.0) and equivalents of H_2O_2 were added to the solution. After each addition of another equivalent of H_2O_2 to the investigated enzyme several minutes were allowed until saturation of the characteristic peak at ~ 345 nm occurred before the next equivalent of H_2O_2 was added. The measurement finished when the characteristic peak (~ 345 nm) did not increase any more with additional H_2O_2. The formation of the *oxy*-form by H_2O_2 has previously been shown for other PPOs,[9-11] however, this is the first study investigating the *oxy*-form formation of mutants in comparison to a wild-type enzyme (*Cg*AUS_{wt}).

Mutant design and visualization of the mutant side chains. For the visualization of the produced mutants the *Cg*AUS_{wt} structure (PDB: 4Z14) was used and the investigated mutants were designed with the molecular visualization system PyMOL. The PyMOL software supplies a number of side-chain orientations (rotamers) for every mutated amino acid residue. Every rotamer is assigned a percentage of possibility according to their frequencies of occurrence in other proteins. In the herein study the highly probable rotamers (according to the PyMOL software) were considered. In this way, the positions of the side chains in the mutants Thr253Asp, The253Asn, Thr253Glu and Arg257Asp were designed and the distances to the adjacent conserved His residues (His252 and His256) were measured. Moreover, the flexibility of the two conserved histidines ($HisA_2$ and $HisB_2$) was investigated in a similar way. The *Cg*AUS_{wt} structure (PDB: 4Z14) and PyMOL were used to depict the different rotamers of the two conserved histidines ($HisA_2$ and $HisB_2$) after release from the dicopper active center due to the flexibility of the copper ions. For the $HisB_2$ the first two rotamers with predicted abundances of ~ 39 and 20 % were considered (from six different possibilities), while for $HisA_2$ only the fourth (from nine different poses as predicted by PyMOL) rotamer with a predicted abundance of 12 % was used as in the first three rotamers only the orientation of the imidazole group was altered, while in the selected rotamer the position of the whole side chain including the Cα-Cβ bond did change.

Random mutagenesis studies by error-prone PCR. Random mutations were introduced into the entire gene of *Cg*AUS_{wt} (minus the signal sequence) by error-prone PCR. The *Cg*AUS_{wt} gene in the expression vector pGEX-6p-1[1] was amplified with the Taq-polymerase (NEB) and the pair of primers for error-prone PCR (Table S2, EP) using an alternative buffer (10 mM Tris-HCl, 50 mM KCl 7 mM, 7 mM $MgCl_2$ and 0.5 mM $MnCl_2$, pH 8.3) in order to increase the error rate of the Taq-polymerase to the required level. The PCR-reaction was limited to 15 cycles only in order create amplicons with ~ 3-7 mutations. One PCR cycle consisted of 20 s at 95 °C, 45 s at 67 °C and 120 s at 68 °C. Prior to the first PCR cycle the reaction was kept at 95 °C for 2 min, after the last cycle a final amplicon elongation step of 8

min at 68 °C was observed. The amplicons were cloned by cut-ligation[12] into the pGEX-6P-SG[13] vector applying the type IIS restriction enzyme Esp3I (NEB) and T4 DNA-ligase (NEB). The resulting plasmids were transformed into chemically competent *E. coli* BL21(DE3) cells. The LB-agar plates used for the culture of the resulting clones did contain an additional auto-induction sugar mix (0.5 % glycerol, 0.05 % glucose and 0.2 % α-lactose) as well as 0.5 mM $CuSO_4$ in order for the target gene to be expressed and the resulting protein to be enzymatically active. From 15 different agar plates ~2000 unique colonies were collected and assayed for monophenolase activity with *L*-tyrosine. The colonies were numbered and resuspended into individual wells of 96-well plates, which contained 200 µl of the assay mixture (100 mM Tris-HCl, 10 mM sodium dodecyl sulfate and 1.4 mM *L*-tyrosine) each. The reaction was monitored for 48 hours at room temperature (circa 23 °C) and color formation was observed for only two out of the 2000 tested colonies. The first monophenolase-positive colony contained three mutations in its *Cg*AUS-gene (**Thr253Ala**, Asp321Asn and Arg385Cys), while the second one carried five mutations (Lys39Arg, **Cys97Ser**, Asn323Ser, Gly372Arg and Ile507Ser) in comparison to *Cg*AUS$_{wt}$. Therefore, the results of this random mutagenesis experiment reinforce the significance of the residues adjacent to the conserved histidines in the type-III copper center as among the numerous investigated colonies only two were able to generate hydroxylase activity and both of them did contain mutations at one of these positions. The *Cg*AUS of the first positive colony had an altered amino acid at the HisB$_1$+1 position (1st activity controller, Thr253Ala) while in the second colony the thioether bridge constituent Cys97 was replaced by serine. The other six mutations from the two colonies are all at least 15 Å away from the active center and are also located on the outer surface of the enzyme. Therefore, we do not expect these mutations to have a pronounced effect on the enzymatic activity of the two positive clones.

Supplementary Notes

Mutations to the gatekeeper residue (Phe273): The gatekeeper residue is usually a phenylalanine in plant PPOs[6,14] but in other organisms (bacteria, fungi and animals) appears as Val,[15] Gly,[16] Ala,[2] Glu[17] and even Thr in the human tyrosinase related protein TYRP1[18]. In *Cg*AUS$_{wt}$, Phe273 has been mutated to Ala, Leu, Asp and His, all resulting in reduced diphenolase activity (Table S1). Notably, the single mutant Phe273Leu exhibited monophenolase activity for tyramine (Table S1 and Figure S4) and *L*-tyrosine (Figure S5). The three mutants Phe273Ala, Phe273His and Phe273Asp were inactive towards monophenols although copper was present in the active site of these mutants (Table S1). All single mutants targeting Phe273 exhibited reduced specificity for dopamine (Table S1) suggesting that Phe at the gatekeeper position stabilizes the phenolic substrate *via* hydrophobic T-shaped *π-π* interactions better than the investigated Ala, Leu, Asp and His amino acid residues.[14,19] However, *π-π* interactions cannot be the basis for monophenolase activity as Phe273Leu exhibits activity with tyramine and *L*-tyrosine (Table S1).

Mutations to the waterkeeper residue (Glu248): This expression refers to a conserved Glu placed at the basis of the active site's cavity in all known PPOs (Figure S1) except for the CO from *Aspergillus oryzae* (*Ao*CO4) which contains the homolog amino acid Gln at this position.[20] Two single mutations, Glu248Ala and Glu248Lys, underlined the significance of the negatively charged amino acids at this position. Glu248Ala reduced the diphenolase activity about 200 times in comparison to *Cg*AUS$_{wt}$, while the Glu248Lys mutant was completely inactive towards all tested substrates, probably due to the lack of copper ions in the active site (Tables S1).

Mutations to the gatekeeper and HisB$_1$+1 (1st activity controller) residues: The double mutant Thr253Asp/Phe273Asp targeting the 1st activity controller and the gatekeeper residue drastically impairs tyrosinase activity, when comparing to Thr253Asp (Table S1). To clarify the impact of the gatekeeper residue in Thr253Asp/Arg257Asp exhibiting the highest monophenolase activity a triple mutant adding Phe273Asp was designed and it shows that the negatively charged Asp at the gatekeeper position blocks mono- and significantly impairs diphenolase activity more than 60 fold (Table S1). Therefore, these results suggest that PPOs exhibit two areas near their active center where an Asp residue influences the tyrosinase activity in opposite directions. Asp positioned at the two activity controllers enhances the hydroxylation of monophenols, while Asp at the gatekeeper position significantly inhibits tyrosinase activity.

Mutations for the displacement of the waterkeeper residue: Two double mutants Glu248Ala/Thr253Glu and Glu248Ala/Phe273Glu were designed in order to move the conserved waterkeeper to the 1st activity controller position and the gatekeeper position, respectively. In Glu248Ala/Thr253Glu the diphenolase activity was reduced 567 times in comparison to the *Cg*AUS$_{wt}$, while monophenolase activity was zeroed (Table S1), whereas in Glu248Ala/Phe273Glu diphenolase activity was reduced 471-fold, while the mutant retained tyrosinase activity with tyramine (k_{cat} = 0.22 s^{-1}, Table S1). Hence, it can be concluded that the conserved waterkeeper residue influences the diphenolase and monophenolase activity when shifted from its original position. Moreover, Glu248Ala/Phe273Glu showed quite similar turnover rates for diphenolase (k_{cat} = 1.18 s^{-1}) and monophenolase (k_{cat} = 0.22 s^{-1}), activities (Table S1), which may indicate that the two activities originate from different structural features of PPOs.

Mutations to the seventh histidine (His285) and the conserved cysteines (Cys31 and Cys32): The His285Ala mutant showed a 38-fold reduced diphenolase activity (Table S1), revealing that the 7th His influences diphenolase activity *via* stabilizing CuB.[21] The mutants (Cys31Ala) and (Cys32Ala) targeting the residues Cys31 and Cys32 which form conserved disulfide bonds (Cys31-Cys94 and Cys12-Cys32, Figure S1) in *Cg*AUS,[14,22] failed to express in *E. coli* (Figure S20). Moreover, the necessity of the loop (Asn98-Ile115) which connects the two residues Cys97 and His116 and form a thioether bridge in *Cg*AUS[14,22] was investigated (Figure S1). The loop (Asn98-Ile115) was deleted and Cys97 was mutated to Leu97. The mutant (deletion loop Asn98-Ile115) was successfully expressed and it was stable in solution, but it showed neither oxidase nor hydroxylase activity, probably due to a deficiency of copper in the active center (Table S1).

3. Supplementary Figures

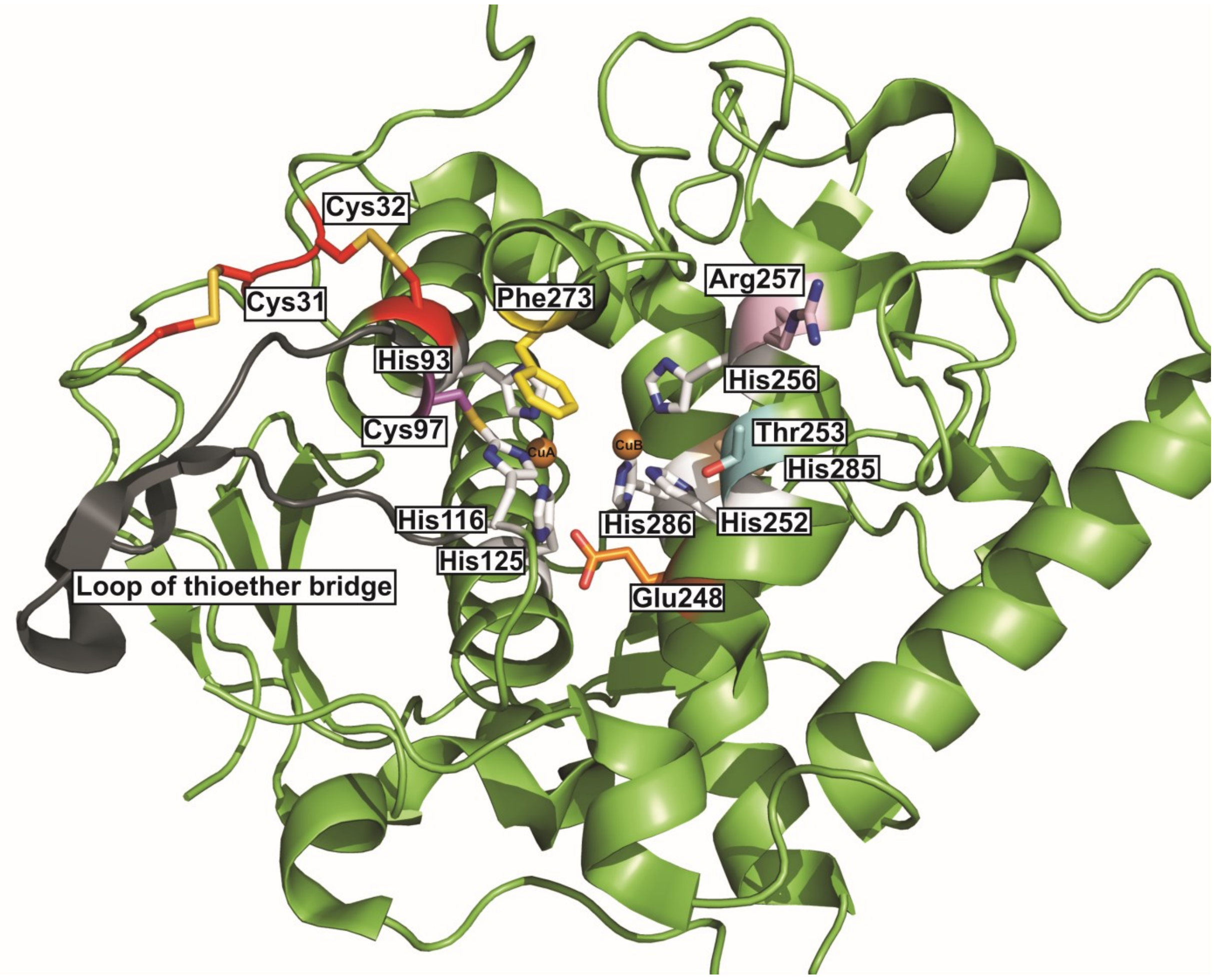

Figure S1. Positions of the mutations and the conserved residues in *Cg*AUS (PDB 4Z14). Mutations toward the following residues were designed; gatekeeper Phe273 (Ala, Leu, Asp and His, yellow), waterkeeper Glu248 (Lys and Ala, orange), the two activity controllers Thr253-HisB$_1$+1 (Asp, Asn, Gly, Ser, Ile, Cys, Glu, Ala and Lys, cyan) and Arg257-HisB$_2$+1 (Leu, Ile, Gly and Asp, light ping) and the thioether bridge constituent Cys97 (Ala, Gly, Asp, Asn and Ser, violet). Moreover, mutations have been performed replacing the 7th histidine His285 (Ala, wheat) and the conserved Cys31 (Ala red) and Cys32 (Ala, red) which form two disulfide bonds respectively. The loop (Asn98-Ile115 deleted) connecting the two residues Cys97 and His116 forming the thioether bridge is shown in black. The six conserved histidines of the dicopper center (CuA: His93, His116, His125 and CuB: His252, His256, His286) are depicted with their carbons highlighted in white and nitrogens in blue.

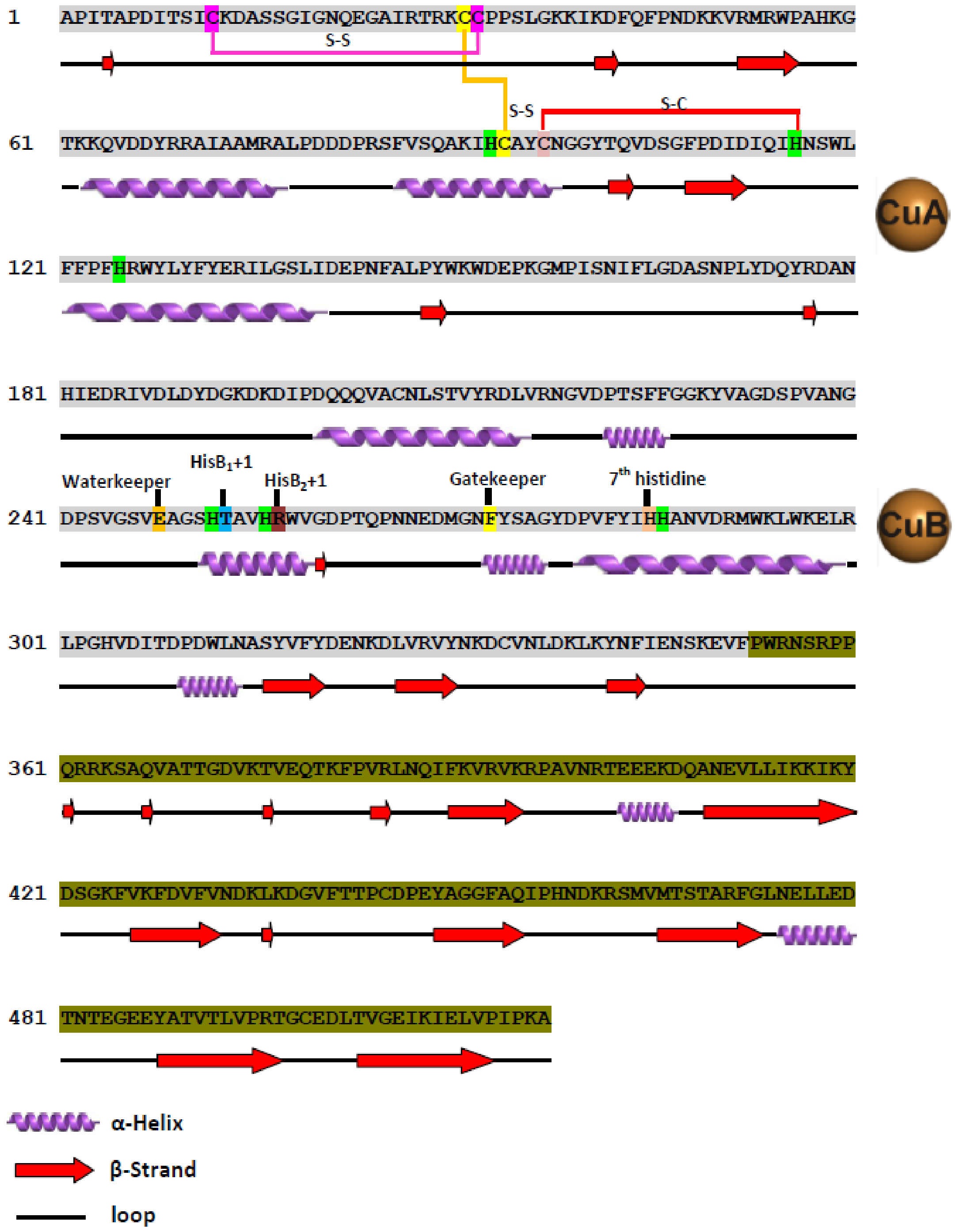

Figure S2. Primary and secondary structure of pro-*Cg*AUS$_{wt}$. The regions forming β-strands (arrows) and α-helices (spirals) are shown next to the sequence of pro-*Cg*AUS$_{wt}$. Additionally, the main domain (grey peptides), the C-terminal domain (olive peptides) and the conserved copper-coordinating histidines (green) of the dicopper center (CuA and CuB) are highlighted. Moreover, the conserved glutamic acid waterkeeper residue Glu248 (orange), the conserved phenylalanine gatekeeper residue Phe273 (yellow), the two amino acids next to the first and the second conserved histidines of CuB HisB$_1$+1 and HisB$_2$+1 (Thr253 cyan and Arg257 violet), the 7th histidine (wheat), as well as the disulfide bonds (S-S) and the thioether bridge (S-C) are also labeled.

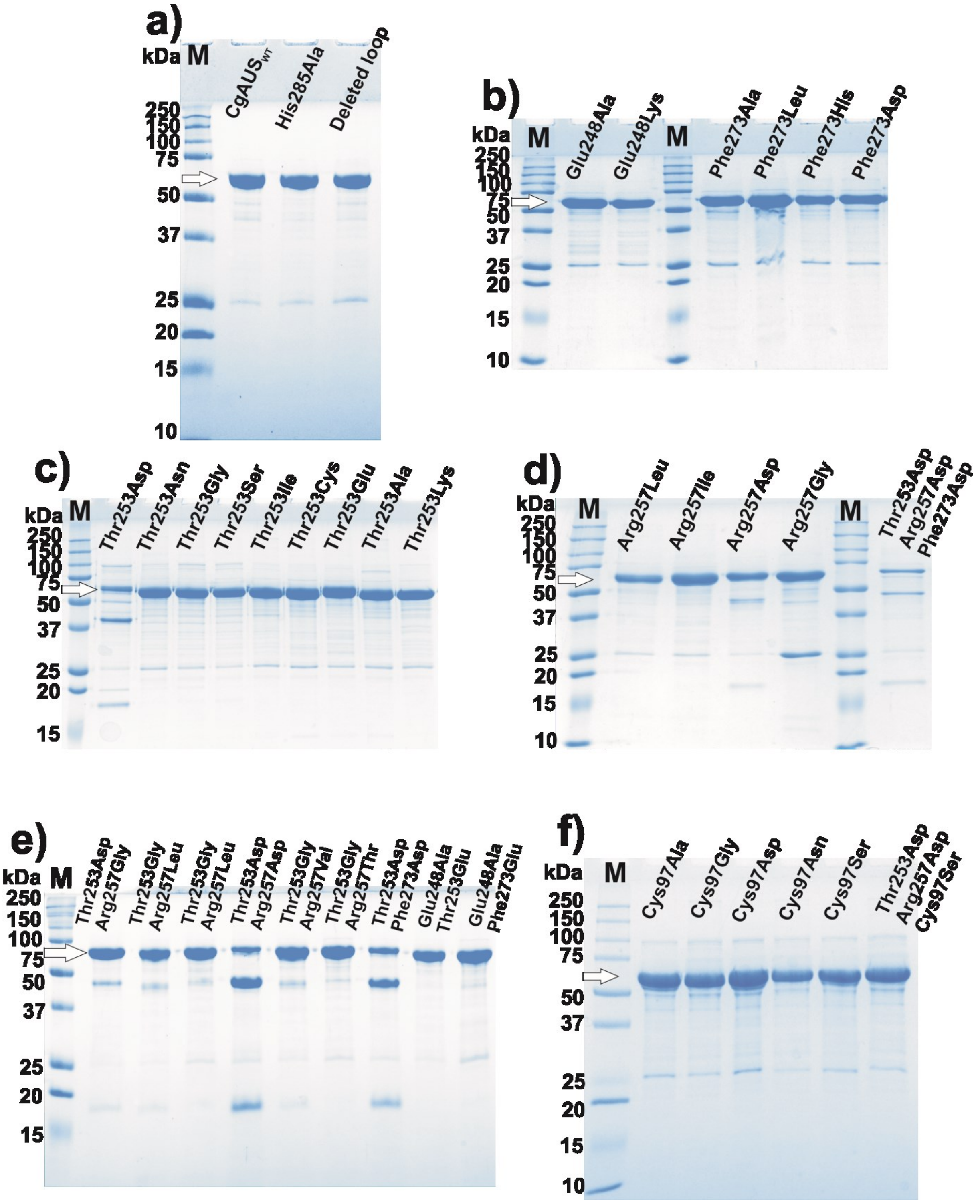

Figure S3. SDS-PAGE gels of the purified *Cg*AUS$_{wt}$ and the investigated mutants. The arrows indicate the positions of the pro-*Cg*AUS$_{wt}$ and the corresponding pro-*Cg*AUS mutants. M: Molecular weight marker in kDa.

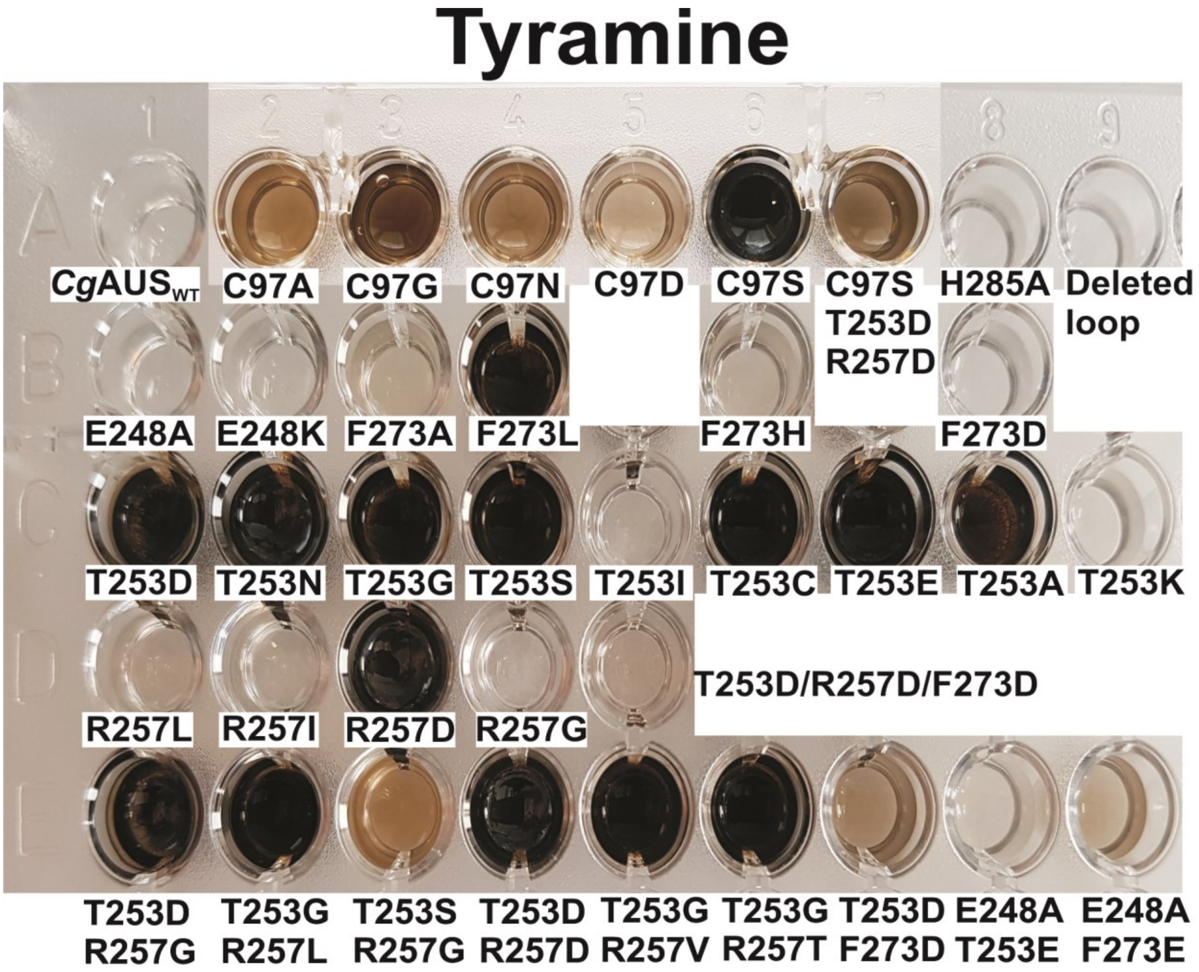

Figure S4. Substrate acceptance assays monitoring the catalytic reactions of *Cg*AUS$_{wt}$ and the investigated mutants with the monophenolic substrate tyramine. Enzymatic reactions were performed in a total volume of 200 µl containing 50 mM Tris-HCl buffer pH 7.0, 50 µg enzyme, 5 mM tyramine and 1.5 mM SDS as activator for 24 h at room temperature. A: alanine, C: cysteine, D: aspartic acid, E: glutamic acid, F: phenylalanine, G: glycine, H: histidine, I: isoleucine, K: lysine, L: leucine, N: asparagine, R: arginine, S: serine, T: threonine, V: valine.

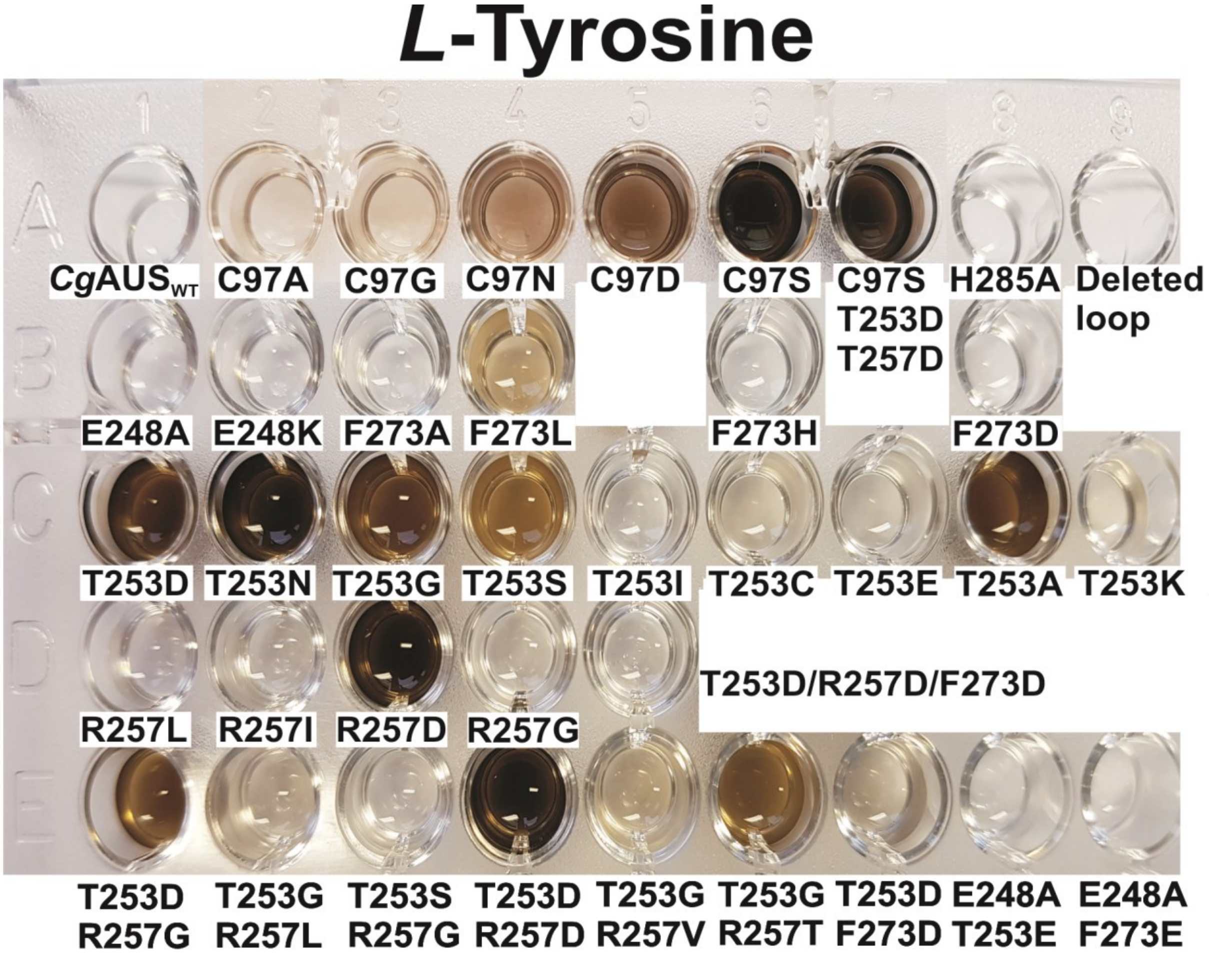

Figure S5. Substrate acceptance assays monitoring the catalytic reactions of *Cg*AUS$_{wt}$ and the investigated mutants with the monophenolic substrate *L*-tyrosine. Enzymatic reactions were performed in a total volume of 200 µl containing 50 mM Tris-HCl buffer pH 7.0, 100 µg enzyme, 1 mM *L*-tyrosine and 1.5 mM SDS as activator for 24 h at room temperature. A: alanine, C: cysteine, D: aspartic acid, E: glutamic acid, F: phenylalanine, G: glycine, H: histidine, I: isoleucine, K: lysine, L: leucine, N: asparagine, R: arginine, S: serine, T: threonine, V: valine.

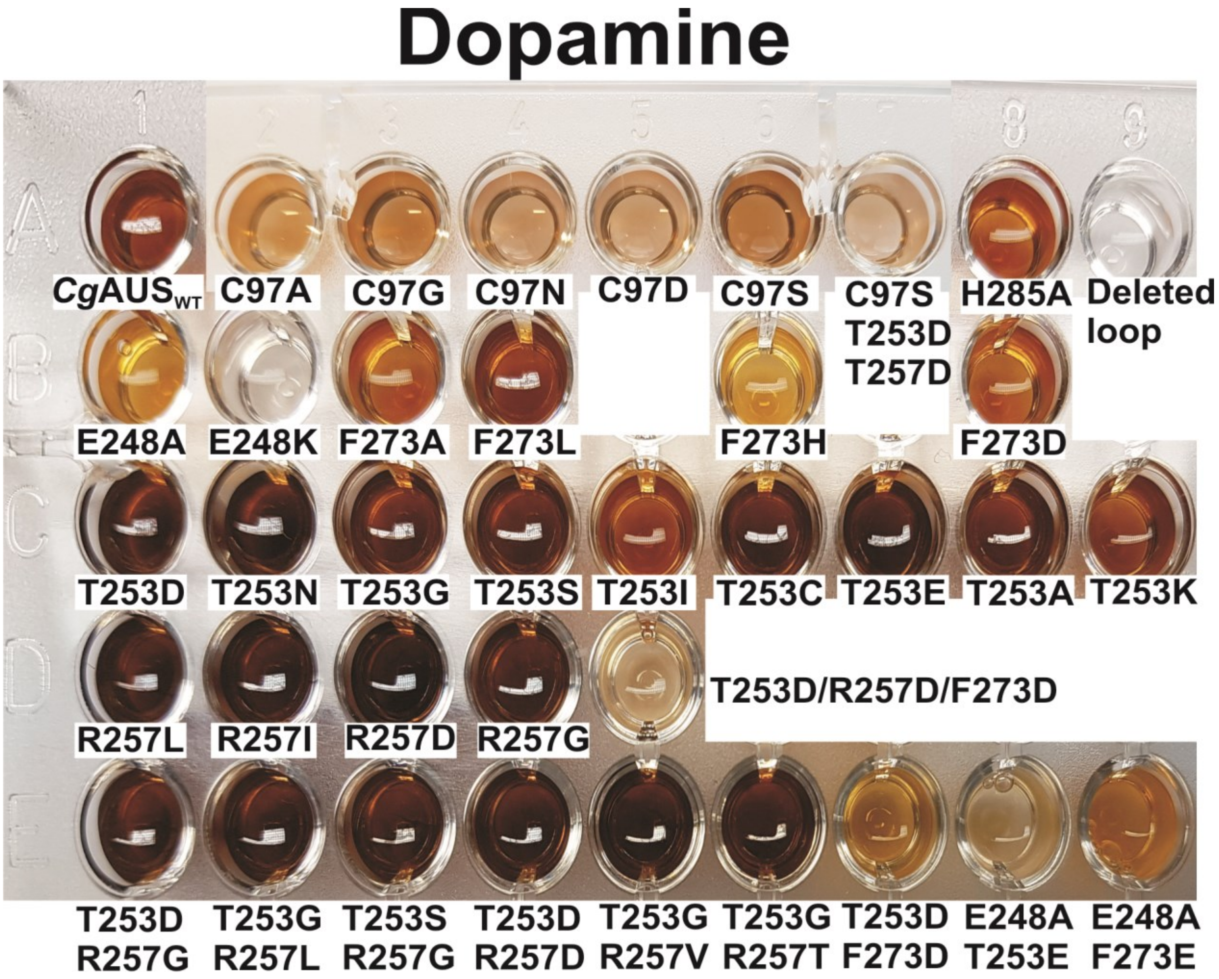

Figure S6. Substrate acceptance assays monitoring the catalytic reactions of *Cg*AUS$_{wt}$ and the investigated mutants with the diphenolic substrate dopamine. Enzymatic reactions were performed in a total volume of 200 µl containing 50 mM Tris-HCl buffer pH 7.0, 5 µg enzyme, 5 mM dopamine and 1.5 mM SDS as activator for 30 min at room temperature. A: alanine, C: cysteine, D: aspartic acid, E: glutamic acid, F: phenylalanine, G: glycine, H: histidine, I: isoleucine, K: lysine, L: leucine, N: asparagine, R: arginine, S: serine, T: threonine, V: valine.

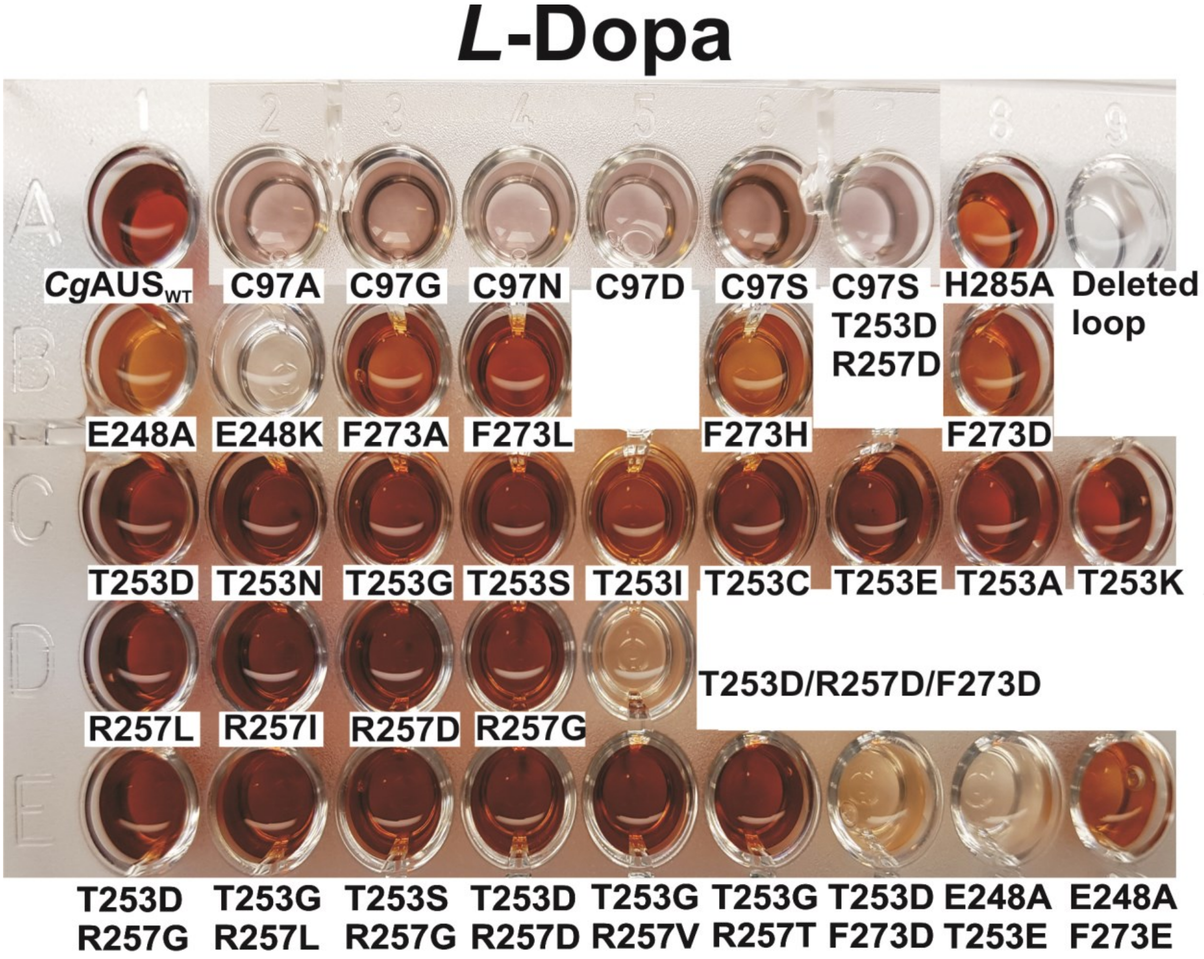

Figure S7. Substrate acceptance assays monitoring the catalytic reactions of *Cg*AUS$_{wt}$ and the investigated mutants with the diphenolic substrate *L*-3,4-dihydroxyphenylalanin (*L*-DOPA). Enzymatic reactions were performed in a total volume of 200 µl containing 50 mM Tris-HCl buffer pH 7.0, 10 µg enzyme, 5 mM *L*-Dopa and 1.5 mM SDS as activator for 30 min at room temperature. A: alanine, C: cysteine, D: aspartic acid, E: glutamic acid, F: phenylalanine, G: glycine, H: histidine, I: isoleucine, K: lysine, L: leucine, N: asparagine, R: arginine, S: serine, T: threonine, V: valine.

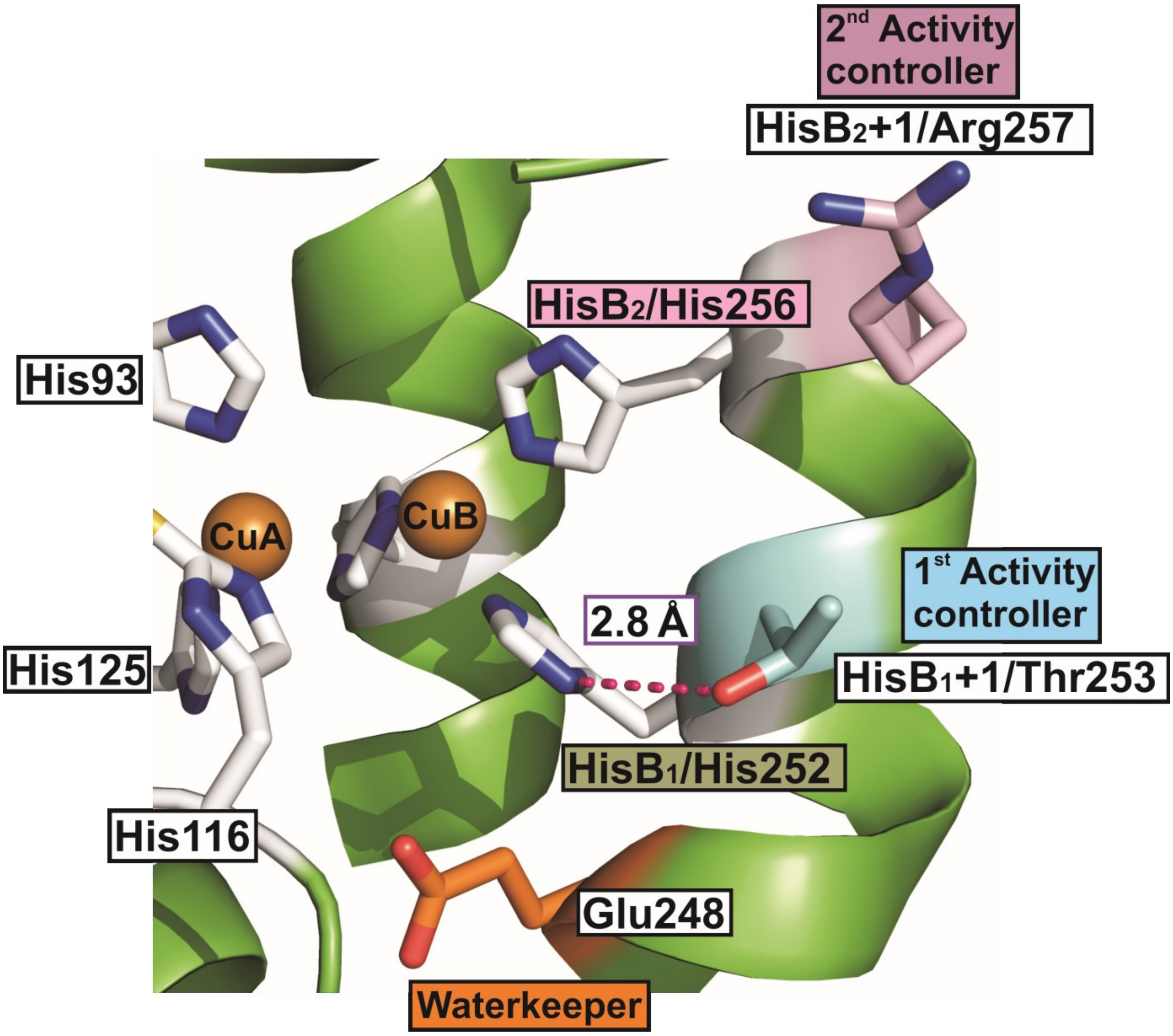

Figure S8: Dicopper active center of *Cg*AUS$_{wt}$ (PDB: 4Z14). The distance between the conserved HisB1 residue (His252) and the adjacent HisB$_1$+1 residue (Thr253, 1st activity controller) is 2.8 Å.

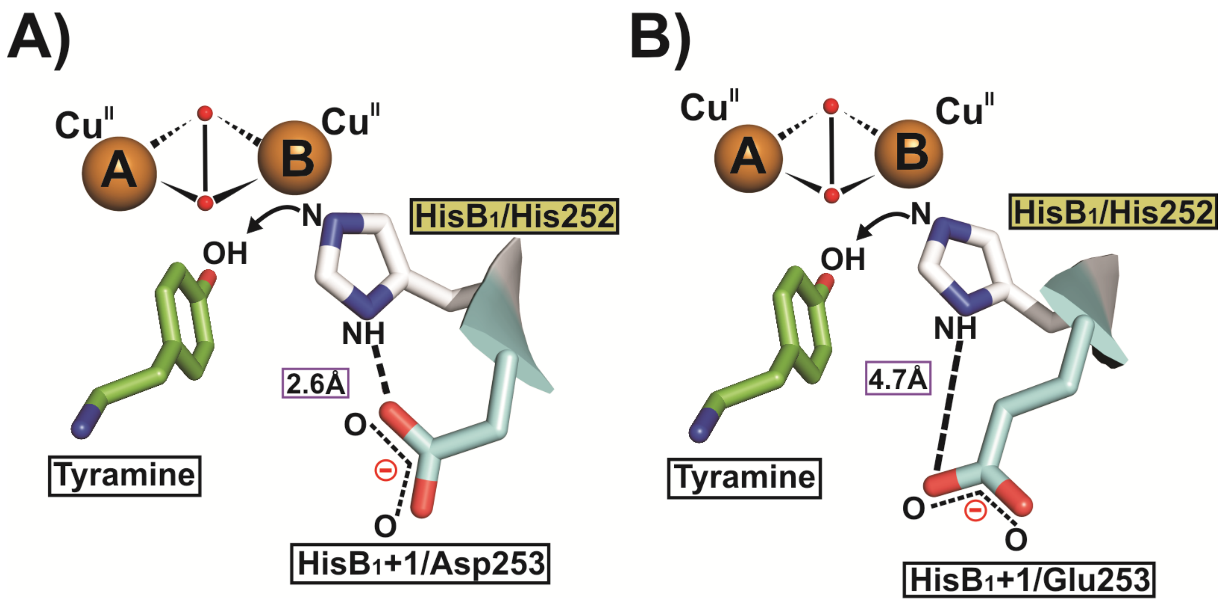

Figure S9: Dicopper active center of Thr253Asp and Thr253Glu mutants. A) Activation of His252 is enhanced by an adjacent negatively charged residue (Asp253) at the $HisB_1$+1 position. The modelled dicopper center of Thr253Asp shows that the distance between Asp253 and His252 residues is 2.6 Å (H_{His252}-O_{Asp253}) which explains the higher activity rate in comparison to Thr253Glu **B)** The dicopper center of Thr253Glu exhibits a distance of 4.7 Å (H_{His252}-O_{Glu253}) between the Glu253 and His252 residues. For the design of the mutants Thr253Asp and Thr253Glu the structure of *Cg*AUS_{wt} (PDB: 4Z14) was used and the amino acid residue Thr253 was replaced by Asp and Glu, respectively. For the position of the site chains the best rotamers as provided by the molecular visualization system PyMOL were used (Asp253 ~61% and Glu253 ~20%). Please find more details in the section "Mutant design and visualization of the mutant side chains" on page 5.

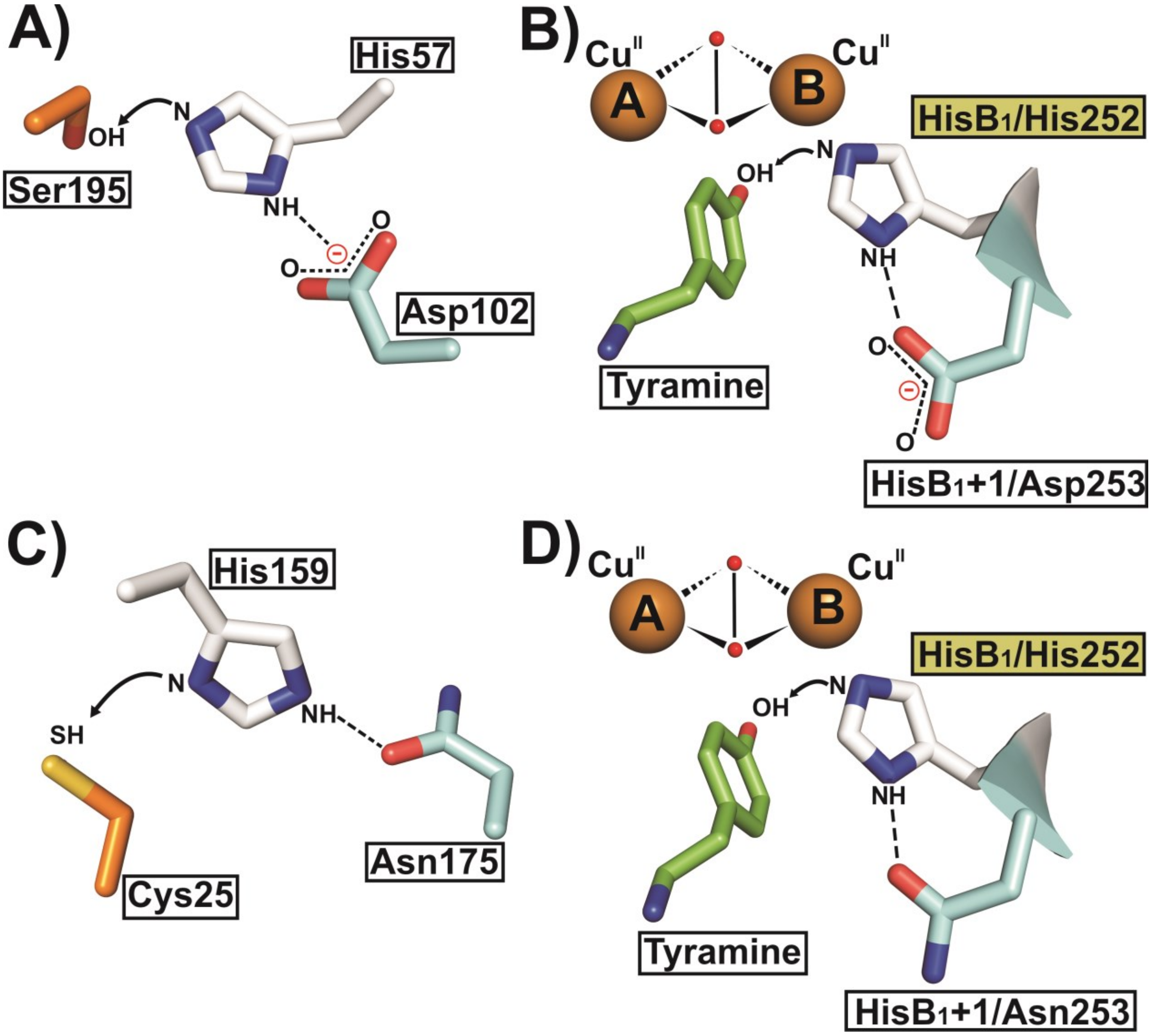

Figure S10: Catalytic triads of chymotrypsin and papain proteases and mimicking combinations of Asp or Asn with His in the Thr253Asp and Thr253Asn mutants. A) Catalytic triad of the serine protease chymotrypsin (PDB: 4H4F) Asp102-His57-Ser195 (acid-base-nucleophile). **B)** Mimicking catalytic complex of the mutant Thr253Asp (Asp253-His252-Tyramine). **C)** Catalytic triad of the cysteine protease papain (PDB: 1PPN) Asn175-His159-Cys25 (stabilizer-base-nucleophile). **D)** Mimicking catalytic complex of the mutant Thr253Asn (Asn253-His252-Tyramine). For the design of the mutants Thr253Asp and Thr253Asn the structure *Cg*AUS$_{wt}$ (PDB: 4Z14) was used and the amino acid residue Thr253 were mutated to Asp and Asn, respectively. For the position of the mutants site chains the best rotamers as provided by the molecular visualization system PyMOL were used (Asp253 ~ 61 % and Asn253 ~ 55 %). Please find more details in the section “Mutant design and visualization of the mutant side chains” (page 5).

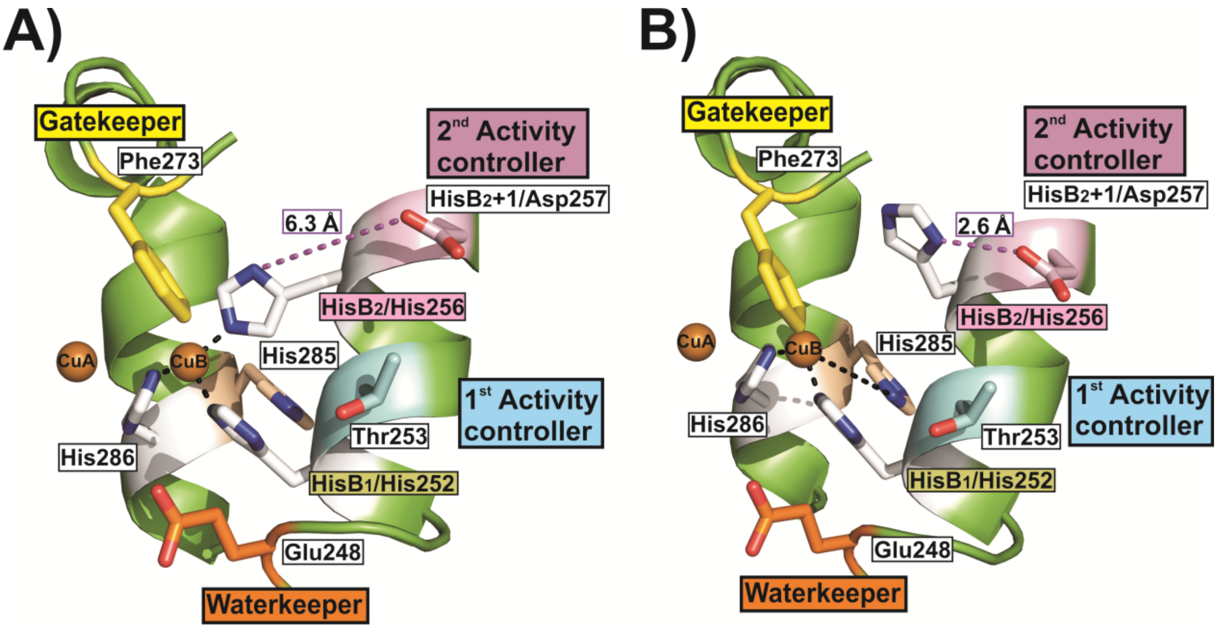

Figure S11. Dicopper active center of the Arg257Asp mutant. A) Represents the interactions of CuB with the three conserved His252, His256 and His286 and shows that the distance between Asp257 ($HisB_2$+1) and His256 ($HisB_2$) is 6.3 Å (H_{His256}-O_{Asp257}). **B)** Represents the release of His256 from the dicopper active center due to the flexibility of the two copper ions and the support of His285 (7th His) towards CuB binding. His256 approaches Asp257 in the Arg257Asp mutant to a distance of 2.6 Å (H_{His256}-O_{Asp257}) and supports the deprotonation of the candidate substrate. For the design of the mutant Arg257Asp the structure *Cg*AUS_{wt} (PDB: 4Z14) was used and the amino acid residue Arg257 was replaced by Asp. For the position of the residue His256 site chain the best two rotamers were used (as provided by molecular visualization system PyMOL: His256 ~ 38 % and 19 %). Please find more details in the section "Mutant design and visualization of the mutant side chains" on page 5.

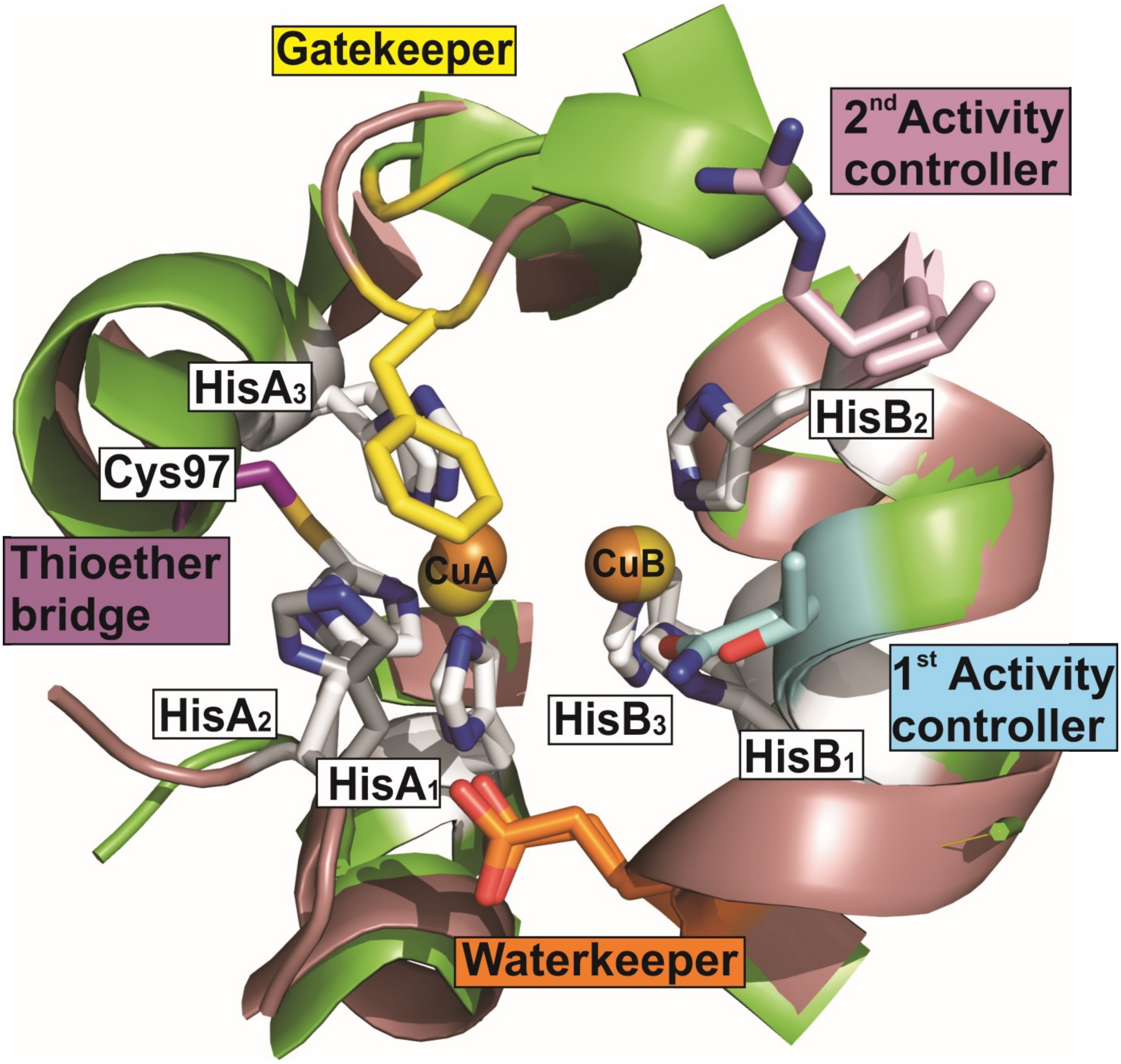

Figure S12. Superimposition of TYR from the bacteria *Streptomyces castaneoglobisporus* (*Sc*TYR, PDB: 3WAT) and plant *Coreopsis grandiflora* AUS (*Cg*AUS, PDB: 4Z14). The figure represents the superimposition of *Cg*AUS$_{wt}$ and *Sc*TYR around their active centers. In *Sc*TYR HisA$_2$ lacks the thioether bridge and reveals a characteristic flexibility as HisA$_2$ (grey and blue) exhibits two different positions in contrast to HisA$_2$ of *Cg*AUS$_{wt}$ which is bound to Cys97 (thioether bridge constituent) and keeps a stable position.

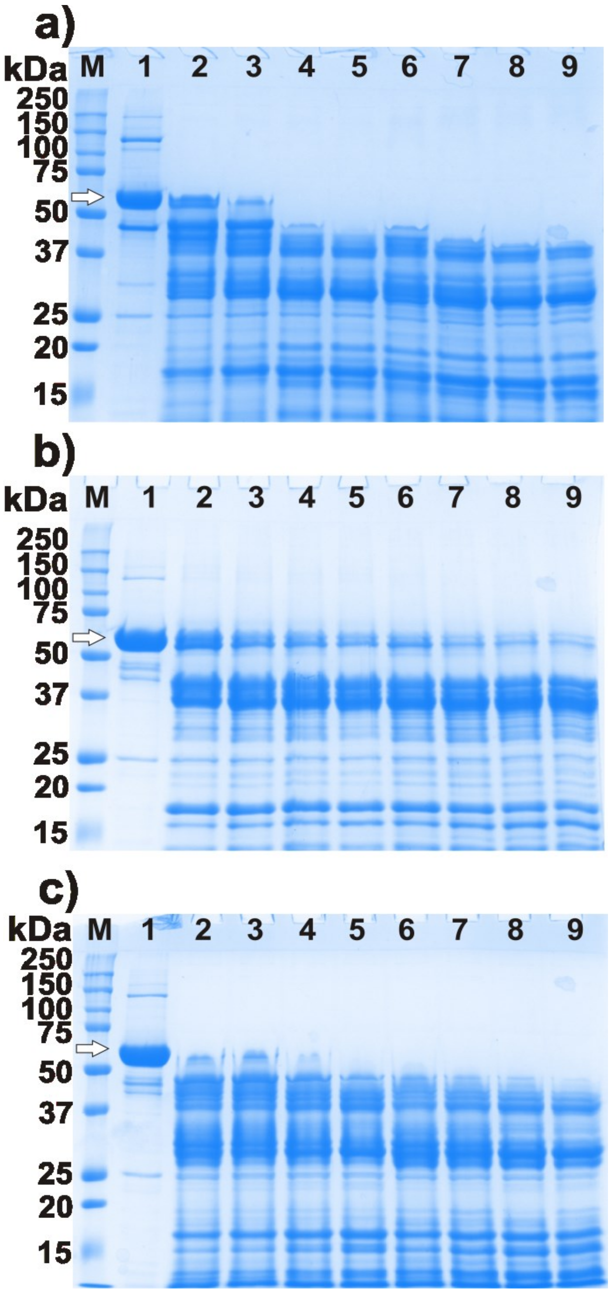

Figure S13. Proteolytic activation of pro-*Cg*AUS$_{\text{wt}}$. SDS-PAGE gels present the proteolytic activation of pro-*Cg*AUS$_{\text{wt}}$ with three different proteases (a) trypsin, (b) proteinase K and (c) nagarse at different mass ratios of protease - protein and different reaction times. Lane 1) *Cg*AUS$_{\text{wt}}$, lane 2) 1:240 (protease:*Cg*AUS$_{\text{wt}}$) for 1 min, lane 3) 1:240 (protease:*Cg*AUS$_{\text{wt}}$) for 2 min, lane 4) 1:240 (protease:*Cg*AUS$_{\text{wt}}$) for 4 min, lane 5) 1:240 (protease:*Cg*AUS$_{\text{wt}}$) for 10min, lane 6) 1:120 (protease:*Cg*AUS$_{\text{wt}}$) for 1 min, lane 7) 1:120 (protease:*Cg*AUS$_{\text{wt}}$) for 2 min, lane 8) 1:120 (protease:*Cg*AUS$_{\text{wt}}$) for 3 min and lane 9) 1:120 (protease:*Cg*AUS$_{\text{wt}}$) for 4 min. The arrows indicate the position of the latent *Cg*AUS$_{\text{wt}}$.

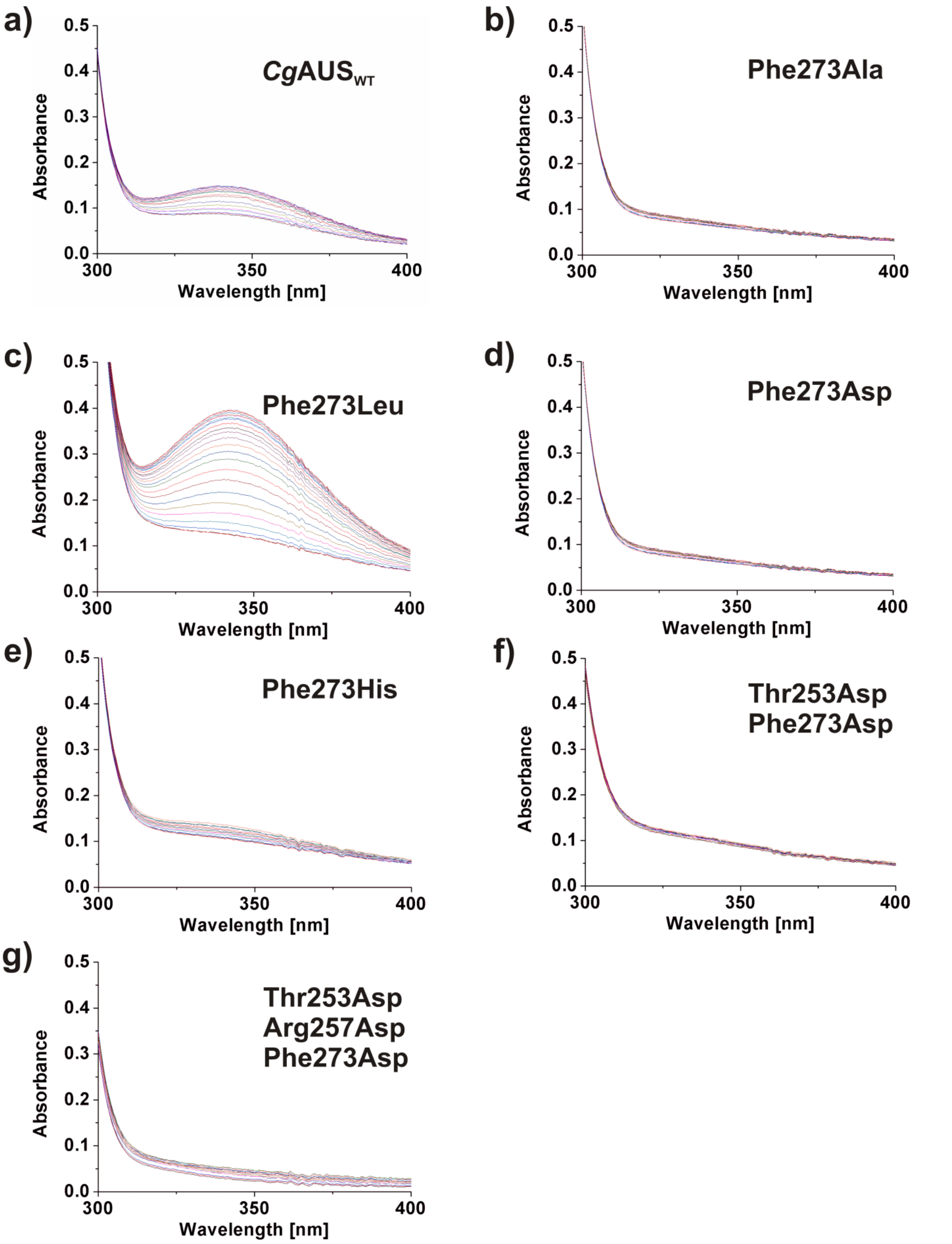

Figure S14. UV/Vis spectra zoomed at 300-400 nm of the *Cg*AUS$_{wt}$ and the gatekeeper residue mutants after treatment with H_2O_2. a) *Cg*AUS$_{wt}$, **b)** Phe273Ala, **c)** Phe273Leu, **d)** Phe273Asp, **e)** Phe273His, **f)** Thr253Asp/Phe273Asp and **g)** Thr253/Arg257Asp/Phe273Asp after treatment with H_2O_2.

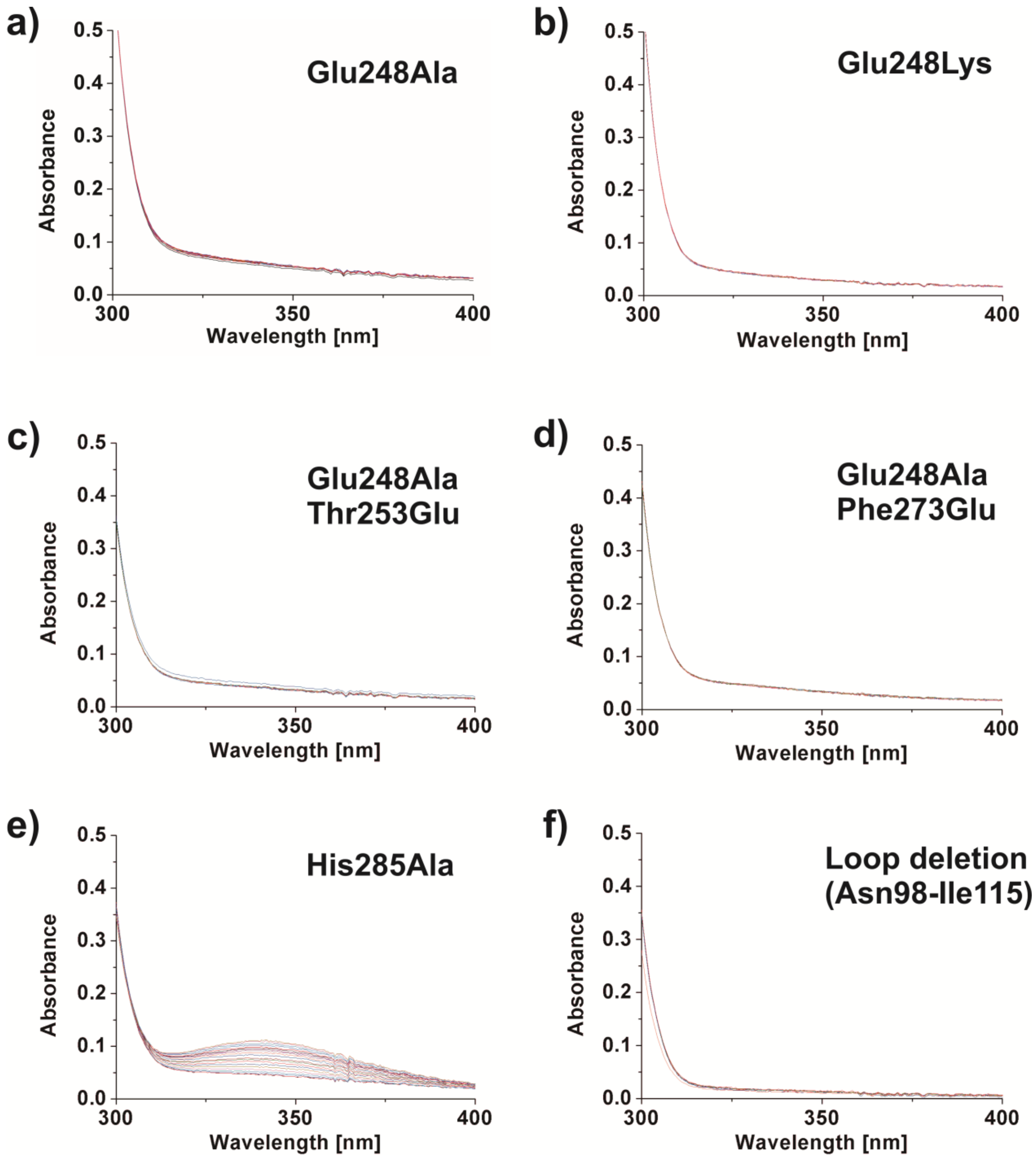

Figure S15. UV/Vis spectra zoomed at 300-400 nm of the waterkeeper residue mutants, the 7th histidine mutant (His285Ala) and the deletion of the loop (Asn98-Ile115) mutant after treatment with H_2O_2. **a)** Glu248Ala, **b)** Glu248Lys, **c)** Glu248Ala/Thr253Glu, **d)** Glu248Ala/Phe273Glu, **e)** His285Ala, and **f)** the loop (Asn98-Ile115) deletion mutant after treatment with H_2O_2.

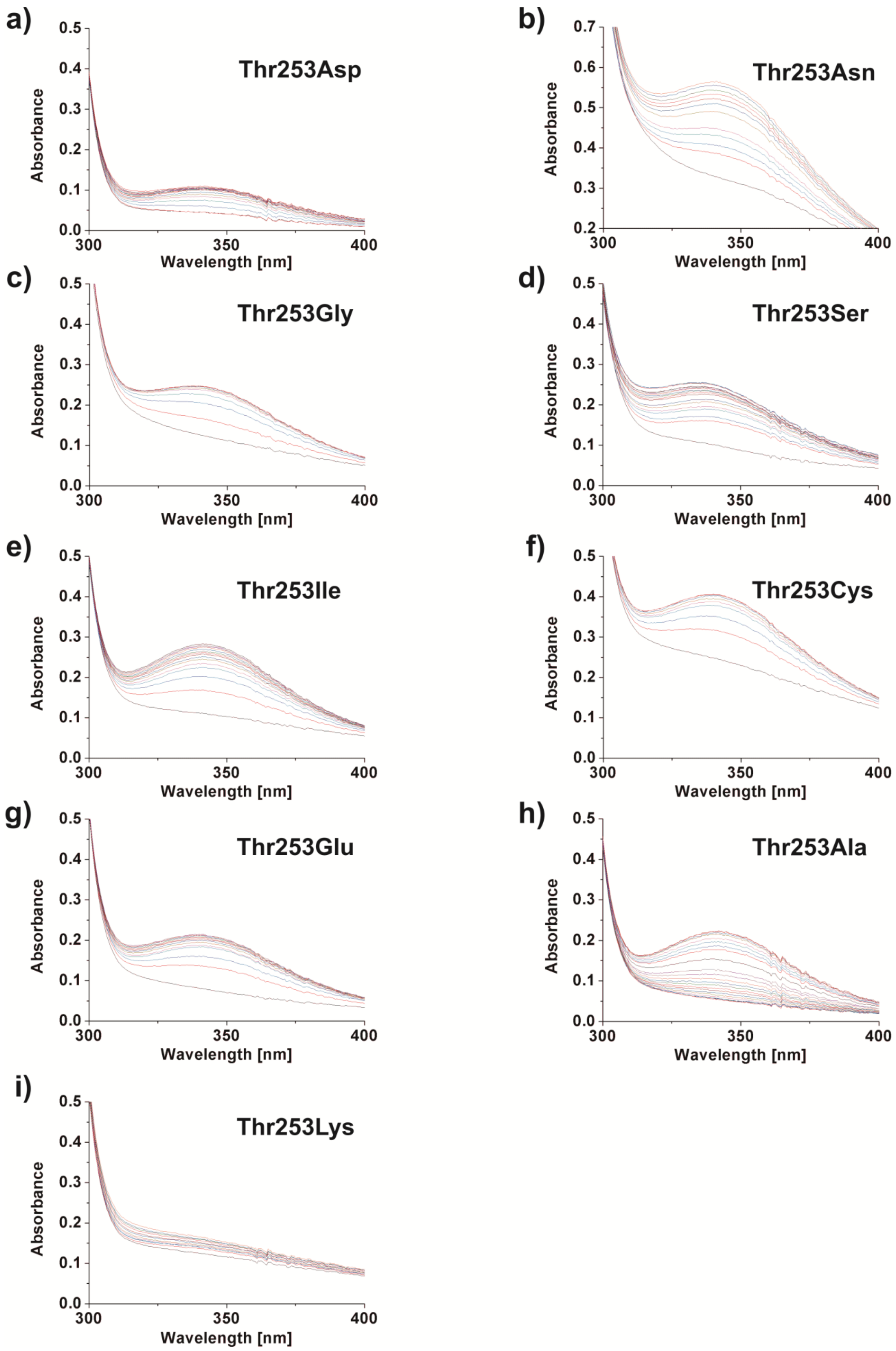

Figure S16. UV/Vis spectra zoomed at 300-400 nm of the HisB$_1$+1 residue (1st activity controller) mutants after treatment with H_2O_2. a) Thr253Asp, **b)** Thr253Asn, **c)** Thr253Gly, **d)** Thr253Ser, **e)** Thr253Ile, **f)** Thr253Cys, **g)** Thr253Glu, **h)** Thr253Ala and **i)** Thr253Lys after treatment with H_2O_2.

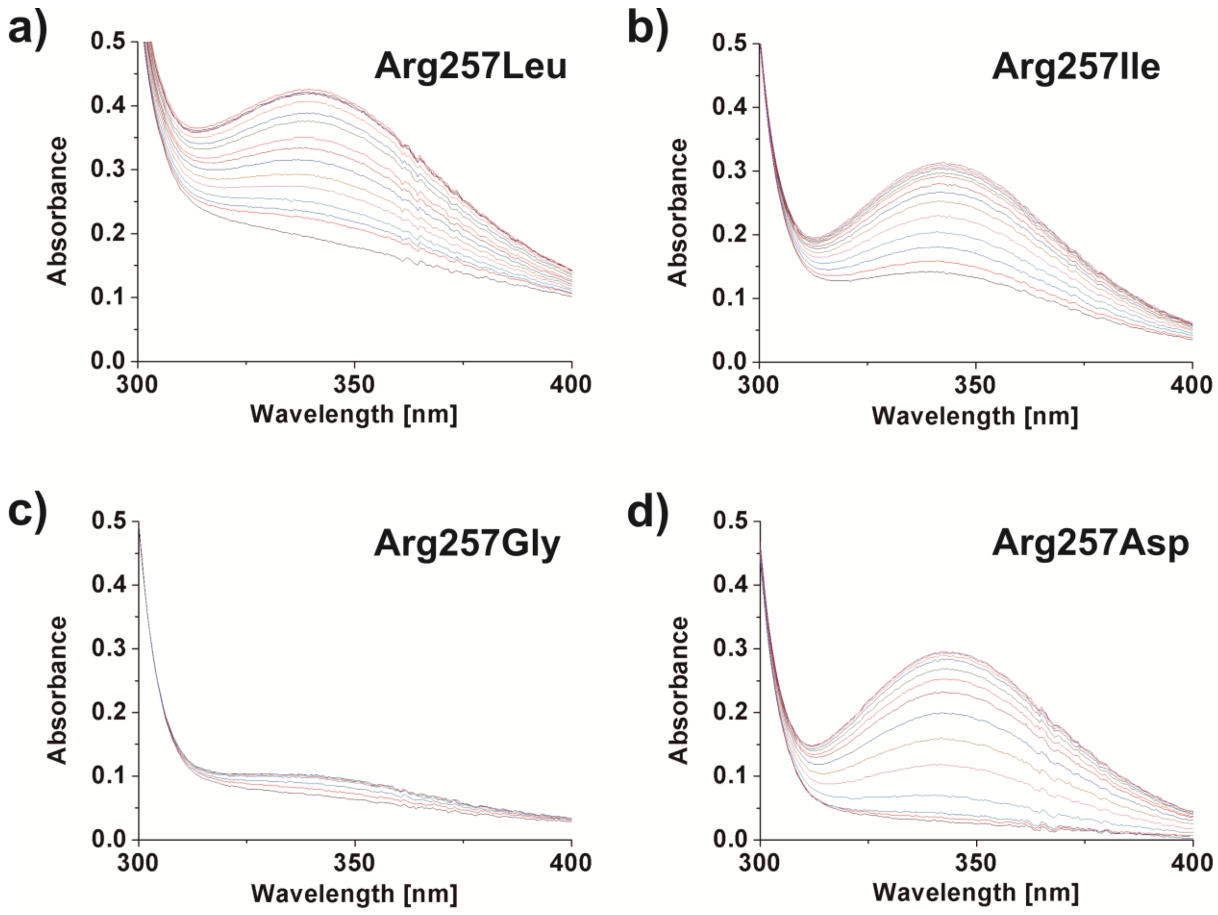

Figure S17. UV/Vis spectra zoomed at 300-400 nm of the HisB$_2$+1 residue (2nd activity controller) mutants after treatment with H_2O_2. **a)** Arg257Leu, **b)** Arg257Ile, **c)** Arg257Gly and **d)** Arg257Asp after treatment with H_2O_2.

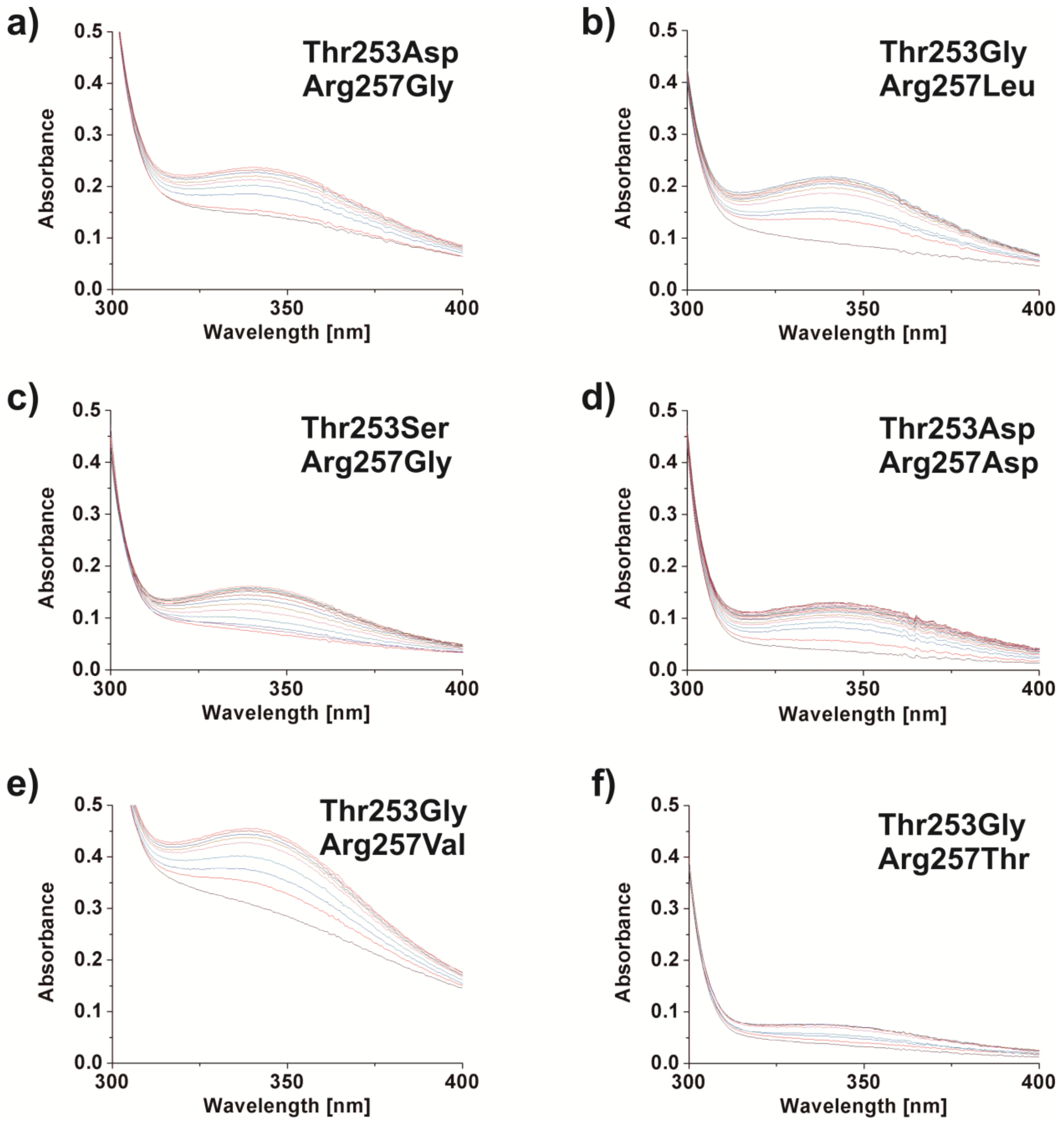

Figure S18. UV/Vis spectra zoomed at 300-400 nm of the double mutants targeting the HisB$_1$+1 and HisB$_2$+1 residues (1st and 2nd activity controllers) after treatment with H_2O_2. **a)** Thr253Asp/Arg257Gly, **b)** Thr253Gly/Arg257Leu, **c)** Thr253Ser/Arg257Gly, **d)** Thr253Asp/Arg257Asp, **e)** Thr253Gly/Arg257Val and **f)** Thr253Gly/Arg257Thr after treatment with H_2O_2.

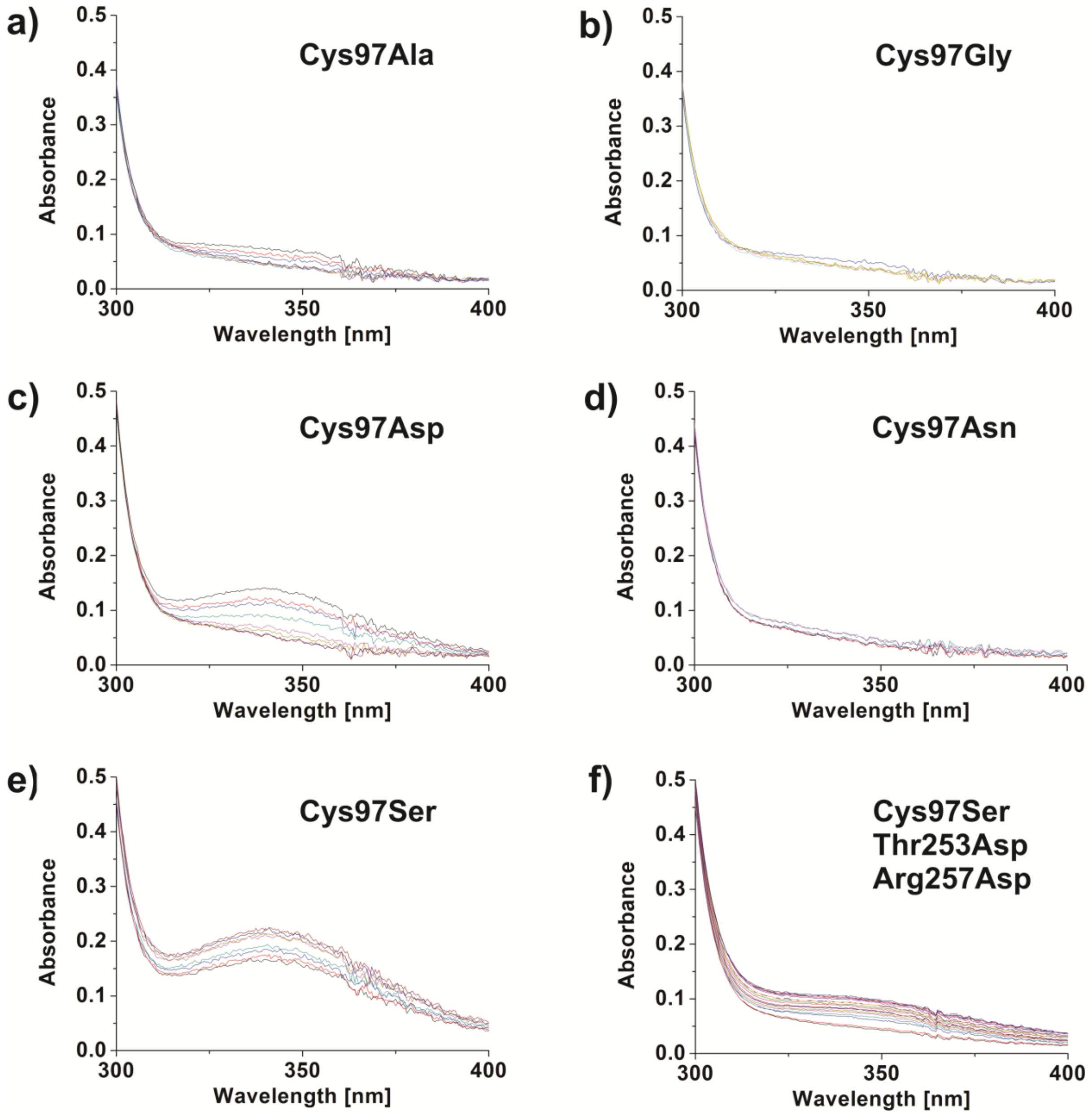

Figure S19. UV/Vis spectra zoomed at 300-400 nm of the thioether bridge constituent mutants after treatment with H_2O_2. **a)** Cys97Ala, **b)** Cys97Gly, **c)** Cys97Asp, **d)** Cys97Asn, **e)** Cys97Ser and **f)** Cys97Ser/Thr253Asp/Arg257Asp after treatment with H_2O_2.

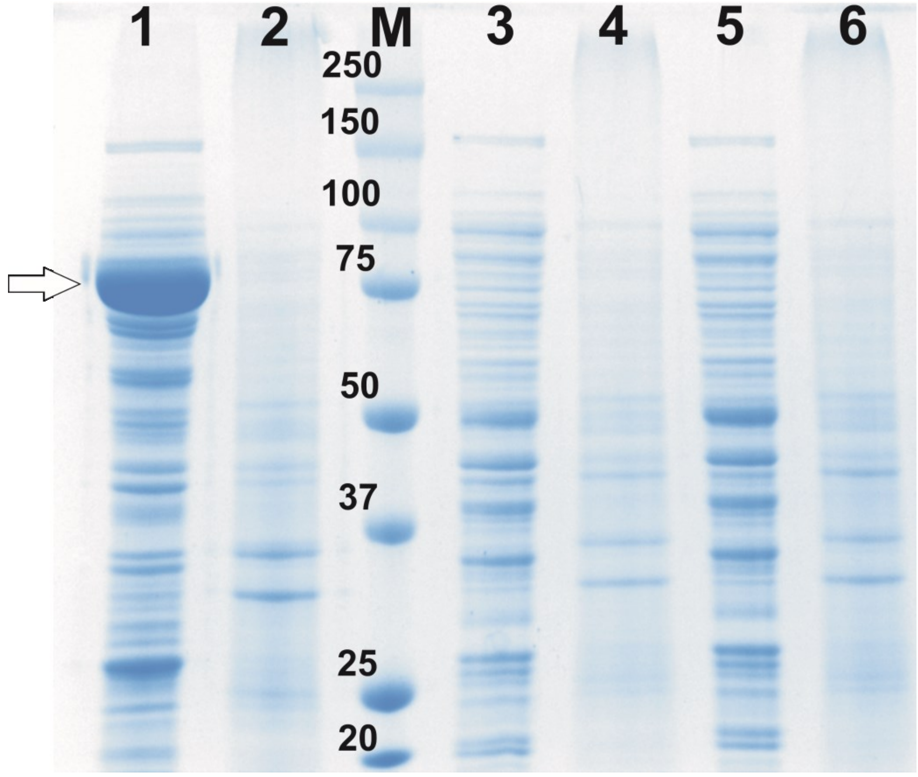

Figure S20. Soluble and insoluble lysate fractions from the heterologous expression of *Cg*AUS$_{wt}$, Cys31Ala and Cys32Ala mutants. Lane **1)** soluble lysate fraction of *Cg*AUS$_{wt}$, lane **2)** insoluble lysate fraction (pellet from the centrifugation step before chromatography, see “Cloning, heterologous expression in E. coli and purification of the CgAUS mutants.”, page 3) of *Cg*AUS$_{wt}$, lane **3)** soluble lysate fraction of Cys31Ala mutant, lane **4)** insoluble lysate fraction of Cys31Ala mutant, lane **5)** soluble lysate fraction of Cys32Ala mutant, lane **6)** insoluble lysate fraction of Cys32Ala mutant. The arrow indicates the position of the expressed target fusion protein (GST-*Cg*AUS). M: Molecular weight marker, in kDa.

Supplementary Tables

Table S1. Measurements of the copper content, k_{cat} and K_m values with dopamine and tyramine, the extinction coefficient ε_{345} at saturation of the mutant-samples with H_2O_2 and the equivalents of H_2O_2 needed for saturation are presented. Mutations that failed to create a peak at 345nm after the incubation with H_2O_2 are presented as "-", while the insoluble mutants are indicated as "ins" and "nd" stands for no detected enzymatic activity or no detected copper.

Mutations	Copper %	Dopamine		Tyramine		H_2O_2 ε_{345} [$M^{-1}cm^{-1}$]	Equiv.
		K_m/mM	k_{cat}/s^{-1}	K_m/mM	k_{cat}/s^{-1}		
$CgAUS_{WT}$	46.1 ± 1.0	8.63 ± 0.28	556 ± 27.2	nd	nd	5190	24
**Gatekeeper residue**							
Phe273Ala	10.1 ± 0.9	9.45 ± 0.71	0.87 ± 0.05	nd	nd	-	-
Phe273Leu	89.5 ± 2.1	15.7 ± 1.27	109 ± 8.50	16.5 ± 2.75	0.27 ± 0.03	14040	60
Phe273Asp	21.3 ± 0.1	9.22 ± 0.76	0.74 ± 0.04	nd	nd	-	-
Phe273His	31.3 ± 0.9	11.3 ± 1.01	9.68 ± 0.69	nd	nd	4660	42
Thr253Asp/Phe273Asp	12.2 ± 1.7	7.31 ± 0.87	1.52 ± 0.11	0.44 ± 0.06	0.01 ± 0.00	-	-
Thr253Asp/Arg257Asp/Phe273Asp	18.1 ± 1.1	4.50 ± 0.41	2.76 ± 0.17	nd	nd	-	-
**Waterkeeper residue**							
Glu248Ala	14.5 ± 0.4	22.5 ± 1.76	2.79 ± 0.20	nd	nd	-	35
Glu248Lys	nd	nd	nd	nd	nd	-	76
Glu248Ala/Thr253Glu	1.2 ± 0.4	13.8 ± 1.24	0.98 ± 0.07	nd	nd	-	106
Glu248Ala/Phe273Glu	1.7 ± 0.1	3.07 ± 0.19	1.18 ± 0.07	41.3 ±15.7	0.22 ± 0.07	-	60
**Thioether bridge constituent**							
Cys97Ala	42.4 ± 1.4	1.59 ± 0.09	5.84 ± 0.31	3.03 ± 0.18	0.14 ± 0.01	-	-
Cys97Gly	34.1 ± 2.3	7.53 ± 0.45	43 ± 2.5	2.96 ± 0.16	0.12 ± 0.01	-	-
Cys97Asp	52.7 ± 0.6	1.68 ± 0.07	1.54 ± 0.07	1.05 ± 0.02	0.07 ± 0.00	-	-
Cys97Asn	45.7 ± 0.6	0.80 ± 0.05	1.24 ± 0.06	2.11 ± 0.08	0.05 ± 0.00	-	-
Cys97Ser	59.3 ± 0.5	1.26 ± 0.04	15 ± 1.26	3.59 ± 0.33	0.55 ± 0.04	7600	18
**$HisB_1$+1 (1st activity controller residue)**							
Thr253Asp	45.0 ± 0.9	1.93 ± 0.12	530 ± 33.7	30.9 ± 7.27	2.14 ± 0.16	3800	48
Thr253Asn	83.6 ± 2.5	4.99 ± 0.38	850 ± 46.5	11.5 ± 2.69	1.19 ± 0.14	19840	21
Thr253Glu	46.7 ± 5.2	3.59 ± 0.22	1394 ± 90.7	11.5 ± 1.06	0.21 ± 0.02	7490	30
Thr253Gly	72.8 ± 0.9	8.74 ± 0.92	337 ± 24.3	2.22 ± 0.14	0.07 ± 0.00	8600	24
Thr253Ser	52.7 ± 0.0	6.62 ± 0.37	500 ± 29.0	1.18 ± 0.09	0.01 ± 0.00	8570	48
Thr253Cys	56.9 ± 1.8	11.1 ± 0.33	312 ± 14.8	4.32 ± 0.28	0.04 ± 0.00	14220	24
Thr253Ala	56.1 ± 3.2	5.80 ± 0.30	140 ± 7.25	1.48 ± 0.04	0.05 ± 0.00	7850	242
Thr253Ile	58.2 ± 0.8	13.6 ± 0.72	27 ± 1.1	nd	nd	9980	43

Thr253Lys	4.8 ± 0.8	38.6 ± 7.88	20 ± 3.2	nd	nd	-	-
**$HisB_2$+1 (2nd activity controller residue)**							
Arg257Asp	11.1 ± 0.8	1.26 ± 0.13	1380 ± 95.3	4.01 ± 0.29	8.26 ±0.48	10470	36
Arg257Leu	64.5 ± 2.1	5.91 ± 0.25	2245 ± 125	nd	nd	14890	48
Arg257Ile	66.3 ± 1.2	3.57 ± 0.17	1660 ± 93.6	nd	nd	11060	40
Arg257Gly	79.6 ± 1.8	3.54 ± 0.26	1264 ± 63.7	nd	nd	3490	18
**$HisB_1$+1 and $HisB_2$+1 (1st and 2nd activity controller residues) and thioether bridge constituent**							
Thr253Asp/Arg257Asp	43.5 ± 1.7	0.24 ± 0.02	171 ± 10.2	1.09 ± 0.09	9.48 ± 0.55	4570	42
Thr253Asp/Arg257Gly	32.8 ± 0.4	1.31 ± 0.04	662 ± 30.0	4.59 ± 0.25	1.91 ± 0.11	8610	27
Thr253Gly/Arg257Leu	33.8 ± 0.8	4.00 ± 0.46	535 ± 39.9	0.75 ± 0.07	0.05 ± 0.00	7690	24
Thr253Ser/Arg257Gly	33.0 ± 0.0	2.37 ± 0.26	430 ± 26.1	0.73 ± 0.10	0.01 ± 0.00	5660	30
Thr253Gly/Arg257Val	52.1 ± 0.4	1.29 ± 0.09	859 ± 52.9	0.46 ± 0.03	0.02 ± 0.00	15990	18
Thr253Gly/Arg257Thr	10.6 ± 1.6	2.09 ± 0.09	191 ± 9.11	15.5 ± 1.03	0.56 ± 0.03	2620	15
Cys97Ser/Thr253Asp/Arg257Asp	40.2 ± 0.6	0.06 ± 0.00	19 ± 1.22	0.02 ± 0.00	6.52 ± 0.39	3700	51
**7th Histidine**							
His285Ala	23.4 ± 0.9	11.2 ± 0.60	14.7 ± 0.84	nd	nd	3850	55
**Deletion of the thioether bridge loop**							
Loop deletion (Asn98-Ile115)	nd	nd	nd	nd	nd	-	-
**Conserved disulphide bonds**							
Cys31Ala	ins	ins	ins	ins	ins	ins	ins
Cys32Ala	ins	ins	ins	ins	ins	ins	ins

Table S2. Primers used for the construction of the *Cg*AUS mutants in pGEX-6P-1.

N	Mutations	Primers	Template
1	***Cg*****AUS$_{WT}$**		
2	**His285Ala**	fw 5´ TTATATCgcCCATGCGAATGTCGACC 3´<br>rev 5´ AACACGGGGTCATACCCA 3´	**Wild type**
3	**Loop deletion of thioether bond**	fw 5´ CACAACTCATGGCTTTTCTT 3´<br>rev 5´taAATAAGCACAATGGATTTTAGC 3´	**Wild type**
4	**Glu248Ala**	fw 5´ GGGTCCGTGGccGCGGGTTCCC 3´<br>rev 5´ GACTGACGGGTCTCCATTC 3´	**Wild type**
5	**Glu248Lys**	fw 5´GGGTCCGTGaaAGCG 3´<br>rev 5´ GACTGACGGGTCTCCATTC 3´	**Wild type**
6	**Phe273Ala**	fw 5´ CATGGGAAACgccTACTCCGCTG 3´<br>rev 5´ TCCTCGTTGTTAGGCTGTG3´	**Wild type**
7	**Phe273Leu**	fw 5´ CATGGGAAACTTgTACTCCGCT 3´<br>rev 5´ TCCTCGTTGTTAGGCTGTG 3´	**Wild type**
8	**Phe273Asp**	fw 5´GGAAACgaCTACTCCGCTGGG 3´<br>rev 5´ CATGTCCTCGTTGTTAGGCTG 3´	**Wild type**
9	**Phe273His**	fw 5´ CATGGGAAACcaCTACTCCGCTG 3´<br>rev 5´ TCCTCGTTGTTAGGCTGTG 3´	**Wild type**
10	**Thr253Asp**	fw 5´ CgatGCCGTGCATAGATGGGTAGG 3´<br>rev 5´ TGGGAACCCGCTTCCACG 3´	**Wild type**
11	**Thr253Asn**	fw 5´ GGTTCCCACAacGCCGTGCAT 3´<br>rev 5´ CGCTTCCACGGACCCG 3´	**Wild type**
12	**Thr253Gly**	fw 5´ GGTTCCCACggGGCCGTGCAT 3´<br>rev 5´ CGCTTCCACGGACCCG 3´	**Wild type**
13	**Thr253Ser**	fw 5´ GGTTCCCACtCGGCCGTGCAT 3´<br>rev 5´ CGCTTCCACGGACCCG 3´	**Wild type**
14	**Thr253Ile**	fw 5´ GGTTCCCACAtcGCCGTGCAT 3´<br>rev 5´ CGCTTCCACGGACCCG 3´	**Wild type**
15	**Thr253Cys**	fw 5´ CtgtGCCGTGCATAGATGGGTAGG 3´<br>rev 5´ TGGGAACCCGCTTCCACG 3´	**Wild type**
16	**Thr253Glu**	fw 5´ GGTTCCCACgaGGCCGTGCAT 3´<br>rev 5´ CGCTTCCACGGACCCG 3´	**Wild type**
17	**Thr253Ala**	fw 5´ GGTTCCCACgcaGCCGTGCAT 3´<br>rev 5´ CGCTTCCACGGACCCG 3´	**Wild type**
18	**Thr253Lys**	fw 5´ GGTTCCCACaaaGCCGTGCAT 3´<br>rev 5´ CGCTTCCACGGACCCG 3´	**Wild type**
19	**Arg257Leu**	fw 5´ CACGGCCGTGCATttATGGGTAG 3´<br>rev 5´ TGGGAACCCGCTTCCACG 3´	**Wild type**
20	**Arg257Ile**	fw 5´ CACGGCCGTGCATatATGGGTAG 3´<br>rev 5´ TGGGAACCCGCTTCCACG 3´	**Wild type**
21	**Arg257Asp**	fw 5´ GTGCATgatTGGGTAGGTGAC 3´<br>rev 5´ GGCCGTGTGGGAACCC 3´	**Wild type**
22	**Arg257Gly**	fw 5´ GTGCATggATGGGTAGGTGAC 3´<br>rev 5´ GGCCGTGTGGGAACC 3´	**Wild type**

23	**Thr253Asp/ Arg257Asp/ Phe273Asp**	fw 5´ GGAAACgaCTACTCCGCTGGG 3´<br>rev 5´ CATGTCCTCGTTGTTAGGCTG 3´	**Mutant 27**
24	**Thr253Asp/ Arg257Gly**	fw 5´GTGCATggATGGGTAGGTGAC 3´<br>rev 5´GGCATCGTGGGAACCC 3´	**Mutant 10**
25	**Thr253Gly/ Arg257Leu**	fw 5´ GTGCATttATGGGTAGGTGAC 3´<br>rev 5´ GGCCCCGTGGGAACCC 3´	**Mutant 12**
26	**Thr253Ser/ Arg257Gly**	fw 5´ CCACtcgGCCGTGCATggATG 3´<br>rev 5´ GAACCCGCTTCCACGGAC 3´	**Mutant 13**
27	**Thr253Asp/ Arg257Asp**	fw 5´ GTGCATgatTGGGTAGGTGAC 3´<br>rev 5´ GGCATCGTGGGAACCC 3´	**Mutant 10**
28	**Thr253Gly/ Arg257Val**	fw 5´ GTGCATgtATGGGTAGGTGAC 3´<br>rev 5´ GGCCCCGTGGGAACCC 3´	**Mutant 12**
29	**Thr253Gly/ Arg257Thr**	fw 5´GTGCATacATGGGTAGGTGAC 3´<br>rev 5´ GGCCCCGTGGGAACCC 3´	**Mutant 12**
30	**Thr253Asp/ Phe273Asp**	fw 5´ GGAAACgaCTACTCCGCTGGG 3´<br>rev 5´ CATGTCCTCGTTGTTAGGCTG 3´	**Mutant 10**
31	**Glu248Ala/ Thr253Glu**	fw 5´CCACGaGGCCGTGCATagATG 3´<br>rev 5´GAACCCGCTgCCACGGAC 3´	**Mutant 16**
32	**Glu248Ala/ Phe273Glu**	fw 5´ GGAAACgagTACTCCGCTGGG 3´<br>rev 5´ CATGTCCTCGTTGTTAGGCTG 3´	**Mutant 4**
33	**Cys31Ala**	fw 5´ GAAAAgcCTGCCCTCCTAG 3´<br>rev 5´ TTGTTCTTATGGCGCCCTC 3´	**Wild type**
34	**Cys32Ala**	fw 5´ GAAAATGCgcCCCTCCTAGC 3´<br>rev 5´ TTGTTCTTATGGCGCCCTC 3´	**Wild type**
35	**Cys97Ala**	fw 5´ TTGTGCTTATgccAACGGTGGGTACACTCAAG 3´<br>rev 5´ TGGATTTTAGCTTGGCTG 3´	**Wild type**
36	**Cys97Gly**	fw 5´ TTGTGCTTATggcAACGGTGGGTACACTCAAG 3´<br>rev 5´ TGGATTTTAGCTTGGCTG 3´	**Wild type**
37	**Cys97Asp**	fw 5´ TTGTGCTTATgacAACGGTGGGTACACTCAAG 3´<br>rev 5´ TGGATTTTAGCTTGGCTG 3´	**Wild type**
38	**Cys97Asn**	fw 5´ TTGTGCTTATaacAACGGTGGGTACACTCAAG 3´<br>rev 5´ TGGATTTTAGCTTGGCTG 3´	**Wild type**
39	**Cys97Ser**	fw 5´ TTGTGCTTATagcAACGGTGGGTACACTCAAG 3´<br>rev 5´ TGGATTTTAGCTTGGCTG 3´	**Wild type**
40	**Cys97Ser/ Thr253Asp/ Arg257Asp**	fw 5´ TTGTGCTTATagcAACGGTGGGTACACTCAAGG 3´<br>rev 5´ TGGATTTTAGCTTGGCTG 3´	**Mutant 27**
EP	**random**	fw 5´ AGCTcgtctcCAATGGCTCCCATAACAGCTCCT 3´<br>rev 5´ AGCTcgtctcATCCCCTAGGCTTTAGGAATAGGAACCAACTC 3´	**Wild type**

References

[1] C. Kaintz, R. L. Mayer, F. Jirsa, H. Halbwirth, A. Rompel, *Febs Lett.* **2015**, *589*, 789–797.

[2] M. Pretzler, A. Bijelic, A. Rompel, *Sci. Rep.* **2017**, *7*, 1810.

[3] E. Gasteiger, C. Hoogland, A. Gattiker, S. Duvaud, M. Wilkins, R. Appel, A. Bairoch, in *Proteomics Protoc. Handb.* (Ed.: J. Walker), Humana Press, **2005**, pp. 571–607.

[4] D. F. Swinehart, *J. Chem. Educ.* **1962**, *39*, 333.

[5] U. K. Laemmli, *Nature* **1970**, *227*, 680–685.

[6] I. Kampatsikas, A. Bijelic, M. Pretzler, A. Rompel, *Sci. Rep.* **2017**, *7*, 8860.

[7] P. M. Hanna, R. Tamilarasan, D. R. McMillin, *Biochem. J.* **1988**, *256*, 1001–1004.

[8] J. L. Muñoz, F. García-Molina, R. Varón, J. N. Rodriguez-Lopez, F. García-Cánovas, J. Tudela, *Anal. Biochem.* **2006**, *351*, 128–138.

[9] A. Rompel, H. Fischer, D. Meiwes, K. Büldt-Karentzopoulos, R. Dillinger, F. Tuczek, H. Witzel, B. Krebs, *J. Biol. Inorg. Chem.* **1999**, *4*, 56–63.

[10] F. Zekiri, C. Molitor, S. G. Mauracher, C. Michael, R. L. Mayer, C. Gerner, A. Rompel, *Phytochem.* **2014**, *101*, 5–15.

[11] I. Kampatsikas, A. Bijelic, A. Rompel, *Sci. Rep.* **2019**, *9*, 4022.

[12] C. Mülhardt, Ed. , in *Exp. Mol. Genomics*, Spektrum Akademischer Verlag, Heidelberg, **2009**, pp. 139–168.

[13] A. Biundo, V. Braunschmid, M. Pretzler, I. Kampatsikas, B. Darnhofer, R. Birner-Gruenberger, A. Rompel, D. Ribitsch, G. M. Guebitz, *Commun. Chem.* **2020**, *3*, 1–8.

[14] C. Molitor, S. G. Mauracher, A. Rompel, *Proc. Natl. Acad. Sci. U. S. A.* **2016**, *113*, E1806–E1815.

[15] M. Sendovski, M. Kanteev, V. S. Ben-Yosef, N. Adir, A. Fishman, *J. Mol. Biol.* **2011**, *405*, 227–237.

[16] Y. Matoba, T. Kumagai, A. Yamamoto, H. Yoshitsu, M. Sugiyama, *J. Biol. Chem.* **2006**, *281*, 8981–8990.

[17] Y. Li, Y. Wang, H. Jiang, J. Deng, *Proc. Natl. Acad. Sci. U. S. A.* **2009**, *106*, 17002–17006.

[18] X. Lai, H. J. Wichers, M. Soler-Lopez, B. W. Dijkstra, *Angew. Chem. Int. Ed.* **2017**, *56*, 9812–9815.

[19] A. Bijelic, M. Pretzler, C. Molitor, F. Zekiri, A. Rompel, *Angew. Chem. Int. Ed.* **2015**, *54*, 14677–14680.

[20] N. Hakulinen, C. Gasparetti, H. Kaljunen, K. Kruus, J. Rouvinen, *J. Biol. Inorg. Chem.* **2013**, *18*, 917–929.

[21] S. G. Mauracher, C. Molitor, R. Al-Oweini, U. Kortz, A. Rompel, *Acta Crystallogr. D Biol. Crystallogr.* **2014**, *70*, 2301–2315.

[22] C. Molitor, S. G. Mauracher, S. Pargan, R. L. Mayer, H. Halbwirth, A. Rompel, *Planta* **2015**, *242*, 519–537.
